# NPF activates a specific NPF receptor and regulates food intake in Pacific abalone *Haliotis discus hannai*

**DOI:** 10.1038/s41598-021-00238-1

**Published:** 2021-10-22

**Authors:** Kyeong Seop Kim, Mi Ae Kim, Keunwan Park, Young Chang Sohn

**Affiliations:** 1 Department of Marine Molecular Biosciences, Gangneung-Wonju National University, 7 Jukheon-gil, Gangneung, Gangwon 25457 Republic of Korea; 2 East Coast Life Sciences Institute, Gangneung-Wonju National University, 7 Jukheon-gil, Gangneung, Gangwon 25457 Republic of Korea; 3 Natural Product Informatics Research Center, KIST Gangneung Institute of Natural Products, Gangneung, Gangwon 25451 Republic of Korea

**Keywords:** Evolution, Molecular evolution, Physiology, Neuroendocrinology, Cell biology, Hormone receptors

## Abstract

Neuropeptides function through G protein-coupled receptors (GPCRs) with high specificity, implying a significant degree of neuropeptide-GPCR coevolution. However, potential neuropeptide signaling systems in non-chordates are relatively elusive. We determined the specificity of the neuropeptide F (Hdh-NPF) signaling system with a cognate receptor (Hdh-NPFR) in the Pacific abalone, *Haliotis discus hannai*. Phylogenetic and exon–intron arrangement analyses of bilaterian NPF and the chordate ortholog NPY with their receptor sequences revealed a likely common ancestor, and Hdh-NPFR was similar to the NPYR2 subtype among the NPYR1, NPYR2, and NPYR5 subtypes. Among four Hdh-NPFR-related receptors, Hdh-NPFR specifically responded to Hdh-NPF peptide, supported by the dose–response luciferase reporter curve, intracellular Ca^2+^ mobilization, and phosphorylation of ERK1/2 and its inhibition with a protein kinase C inhibitor. Peptide fragmentations and shuffling of Hdh-NPF with human NPY could not activate the cellular response of Hdh-NPFR. Three-dimensional in silico modeling suggested that interaction of Hdh-NPF C-terminal amino acids with the extracellular loops of Hdh-NPFR is critical for Hdh-NPFR activation. In vivo injection of Hdh-NPF peptide increased food consumption, and knockdown of *Hdh-NPF* expression decreased food consumption in Pacific abalone. These findings provide evidence for co-evolution of the NPF/Y ligand-receptor system, enabling further research on mollusk orexigenic neuropeptides.

## Introduction

Neuropeptides (NPs) synthesized and secreted by neurons are essential regulators in diverse animal phyla. NPs exert their effects locally as hormones, neurotransmitters, and neuromodulators for various physiological functions, including growth, metabolism, and reproduction^1–3^. The evolutionary origins of NPs have been traced to the common ancestor of protostomes and deuterostomes, in which two rounds of genome duplication in the vertebrate lineage gave rise to an expanded repertoire of NP signaling systems with cognate receptors^4^. For example, identification of the [LV]Wamide ([LeuVal]Trp-NH_2_) cluster in the eumetazoan species has shed light on the complex mechanism of duplication and gene loss for the origin of these NP families, such as the bilaterian adipokinetic hormone (AKH) and gonadotropin-releasing hormone (GnRH)/corazonin (CRZ) families^5^. Given the conserved role of GnRH in reproductive endocrine systems, lineage-specific loss of CRZ signaling in vertebrates and duplication of the GnRH signaling system with AKH in invertebrates may account for the global phylogenetic distribution of AKH/CRZ/GnRH-type receptor pathways^6^.

Neuropeptide Y (NPY), a 36-amino acid (aa) peptide originally isolated from the porcine brain^7^, belongs to the NPY family of biologically active peptides, together with two other members: peptide YY (PYY) and pancreatic polypeptide (PP)^8^. In vertebrates, NPY plays an essential role in diverse physiological functions, including food intake, energy homeostasis, anxiety, and stress responses, through interaction with NPY receptors (NPYRs)^9–11^, which belong to class A G-protein coupled receptors (GPCRs), activated by the closely related peptides NPY, PYY, and PP^12^. Activation of vertebrate NPYRs by NPY primarily results in decreased cyclic adenosine monophosphate (cAMP) accumulation through Gi protein, leading to the inhibition of adenylyl cyclase in mammalian cells^12^. Furthermore, human NPY-activated NPYRs induced increases in intracellular Ca^2+^ levels possibly through Gq protein^13,14^. Recognition experiments of mammalian NPY by its receptors using chemically modified NPYs, receptor mutagenesis, and receptor chimeras demonstrated that NPYRs require both ends of NPY for binding, and intact Tyr-32, Arg-35, and Tyr-36-NH_2_ residues are critical for the fully active form^15,16^. Small-molecule compounds that regulate NPY signaling systems have pharmaco-therapeutic implications, with potential efficacy in the treatment of a wide range of metabolic and psychiatric disorders^17,18^.

Invertebrate NPF, an ortholog of human NPY terminating in a Phe-amide, and NPF-related NPs (e.g., sNPF, FLP-34, and PrRP) exert their effects on target cells by binding to and activating specific types of receptors, leading to changes in the activity of downstream effectors^19–22^. Since discovery of the first invertebrate NPF in the tapeworm *Monieza expansa*^23^, several NPF orthologs that typically display an RPRF-NH_2_ C-terminal sequence and a length ranging from 36 to 40 aa have been identified from diverse invertebrate phyla^20^. However, functional studies for NPF signaling systems with NPFRs and their related receptors have mainly focused on model organisms, especially *Drosophila* and *Caenorhabditis*^19–21^. Invertebrate NPF systems, including sNPF, seem to play crucial roles in the coordination of feeding, energy metabolism, circadian rhythm, and reproduction^20,24–26^. Recent findings offer new insights into the roles of the *Drosophila* NPF signaling pathway in male courtship and germline stem cell proliferation^27,28^.

Mollusca is the second-largest phylum of living animals, with an estimated 85,000 extant species of mollusks comprising approximately 23% of all named marine animals^29^. Although the first functional invertebrate NPFR was characterized from the brain of the snail *Lymnaea stagnalis*^30^, there is limited and controversial information on the in vivo effects of molluscan NPFs. Injection of NPF increased the filtration rates of clams (*Ruditapes philippinarum*)^31^, whereas *Aplysia californica* NPF was found to reduce food intake in a dose-dependent manner^32^. Further, administration of *L. stagnalis* NPF to the snail did not have short-term effects on food intake, but reduced growth and reproductive performance^33,34^. To fully explain these elusive NPF functions, it is essential to investigate the distribution, spatiotemporal expression pattern, and functional characterization of NPF signaling systems in non-model mollusks. Since the *L. stagnalis* NPFR is the only known functional receptor interacting with molluscan NPF identified to date^30^, characterization of additional receptors is needed to better understand the possible roles of NPF in mollusks.

Abalone (Mollusca: Gastropoda: Haliotidae) farms provide high-quality seafood for humans. Recently, farm production of this species has increased from negligible quantities, with the vast majority being produced in China and South Korea at 87% and 10% respectively, according to worldwide production records^35^. However, environmental and farm stress imposes changes to abalone physiology and metabolism, such as reductions in juvenile growth rates and spawning, threatening the global and sustainable aquaculture industry of abalone^36^. To better understand the reproductive mechanism in the Pacific abalone *Haliotis discus hannai* (Hdh), we recently analyzed the neural ganglia transcriptome and peptidome associated with sex, growing stage, and sexual maturation of this species^37,38^. Given that NPF/Y signaling is known to regulate food intake in both vertebrates and invertebrates^9,10,20^, we hypothesized that Hdh-NPF regulates food intake in Pacific abalone. We here report the identification and characterization of a functional Hdh-NPF signaling system. The novel molluscan Hdh-NPFR can provide further insights into the evolution and function of NPF/Y receptors across bilaterian organisms.

## Results

### Sequence analysis of Hdh-NPF precursors

**Figure 1:**
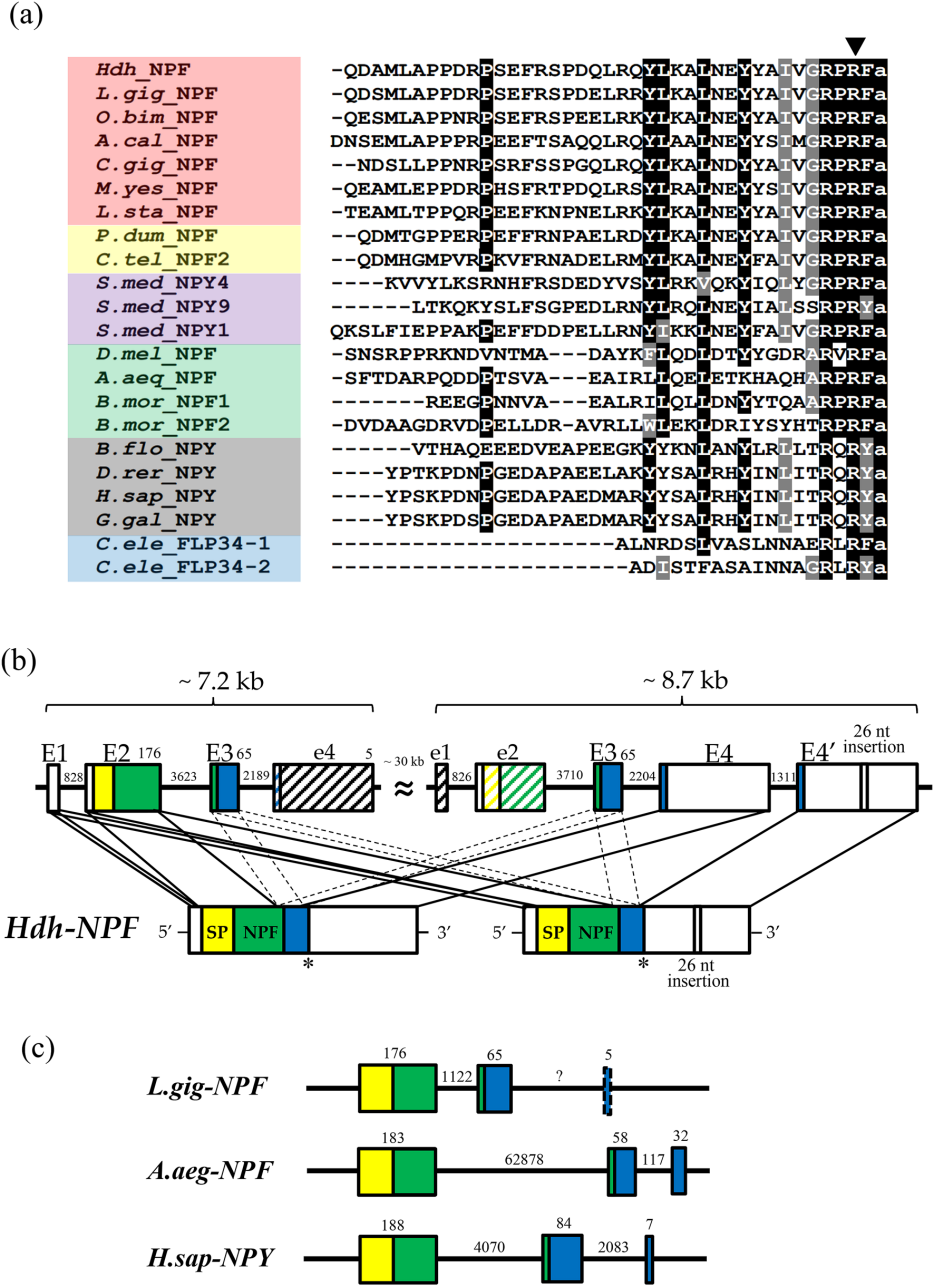
NPF and NPY peptides with *H. discus hannai* (*Hdh*) *NPF* genomic structure. (**a**) Comparison of the amino acid sequence of Hdh-NPF peptide with those of related NPF/Y peptides from other phyla. Black and grey shaded amino acids represent identical and similar residues, respectively, with a minimum of 70% conservation. Exon–intron boundary is represented by the downward solid arrowhead. The color coding of phyla is as follows: red (Mollusca), yellow (Annelida), violet (Platyhelminthes), green (Arthropoda), grey (Chordata), blue (Nematoda). The full name of the species and the accession numbers of the sequences are listed in Supplementary Table S1. (**b**) Schematic representation of two *Hdh-NPF* transcripts with corresponding *NPF/NPY* genes in the *Hdh* genome (accession number PRJNA317403) and (**c**) *Lottia gigantea* (*L.gig*; NW_008710370.1), *Aedes aegypti* (*A.aeg*; NC_035107.1), and *Homo sapiens* (*H.sap*; NG016148) genomes. Signal peptides (SP), the mature NPF/Y, and the NPF/Y-associated peptide are shown by yellow, green, and blue boxes, respectively, with 5′- and 3′-untranslated regions shown in white boxes. Exons (E1–E4′) and introns are indicated by boxes and lines, respectively. Dotted lines linked to two E3 represent undefined joining of E3 between E2 and E4/E4′. Hatched boxes labeled with e1, e2, and e4 indicate highly similar sequences to E1, E2, and E4, respectively, but have different reading frames from *Hdh-NPF,* because of frameshift mutations. Numbers on boxes and lines indicate the length of nucleotides.

The lengths of the nucleotide sequences predicted to be Pacific abalone *NPF* transcripts (*Hdh-NPF*) were 621 bp and 647 bp, including a 5′-untranslated region (UTR), 3′-UTR, and poly(A) tail (Supplementary Fig. S1). Two *prepro-Hdh-NPF* transcripts had identical sequences, except for the 26-nucleotides insertion in the transcript with a longer splicing variant. The coding sequences of the *prepro-Hdh-NPF* transcripts encoded 81 aa, including a 21-aa signal peptide and a 39-aa mature Hdh-NPF peptide. A cleavage site was identified at the end of mature Hdh-NPF with a G residue responsible for biological amidation. Mature NPF/Y peptide sequences from representative species in the phyla Mollusca, Annelida, Platyhelminthes, Arthropoda, Chordata, and Nematoda were compared as shown in Fig. 1a. RPRF in the C-terminal and P8,11 residues, which are known to be important for NPY receptor affinity^39^, were well conserved among the NPF/Y orthologs. Further mapping of the *Hdh-NPF* gene revealed that two *prepro-Hdh-NPF* precursors were transcribed from two different *Hdh-NPF* genes (Fig. 1b), which are on the same scaffold in a stretch of 45.9 kb^40^. Approximately 7.2 and 8.7 kb-long *Hdh-NPF* genes comprised 4 exon–3 intron and 5 exon–4 intron structures, respectively, and shared a relatively high sequence similarity (63.7%). The nucleotide sequence identities between two *Hdh-NPF* genes of exon 2, 3, and 4 were 91%, 100%, and 96%, respectively. The first and second exons (E1 and E2) of the shorter 7.2 kb-long gene commonly encoded 5′-UTRs, signal sequences, and the majority of Hdh-NPF peptide regions in two *prepro-Hdh-NPF* precursors, whereas the last two exons (E4 and E4′) of the 8.7 kb-long gene were alternatively spliced for the two transcripts (Fig. 1a,b). The second intron located between the second and the third nucleotide of the second R codon in the C-terminal RxRF/Yamide motif of Hdh-NPF peptide was strictly conserved with the corresponding exon–intron borders in other vertebrate *NPY* and invertebrate *NPF* genes (Fig. 1a,c). Using the maximum likelihood method, a phylogenetic analysis was performed with bilaterian NPF/Y, invertebrate deuterostome sNPF/PrRP precursors, and protostome sNPF precursors, along with two molluscan GnRH precursors as outgroups. This showed that the aa sequence of the Hdh-NPF precursor was grouped with those of the molluscan NPF precursors into a lophotrochozoan NPF subfamily (Supplementary Fig. S2).

### Sequence analysis of Hdh-NPFRs

Four putative abalone NPF receptors (Hdh-NPFRs) were identified by a BLAST search with *L. stagnalis* NPFR (*L.sta*_NPFR or *L.sta*_GPCR105) and human NPYRs. Among them, we named the NPFR with the highest aa sequence identity (57.4%) to *L.sta*_NPFR as Hdh-NPFR (373 aa) and the others were designated Hdh-NPFR-like-1 (419 aa), -2 (377 aa), and -3 (390 aa) in the order of sequence similarity (27.4–22.9%). The abalone receptors had one N-terminal extracellular domain (ECD), seven trans-membrane domains (TMDs), three extracellular and intracellular loops (ECLs and ICLs), and one C-terminal intracellular domain (ICD). We also determined two potential *N*-glycosylation sites in the ECD and a characteristic E/DRY/F sequence of rhodopsin-like GPCR in the second ICL of Hdh-NPFR (Supplementary Fig. S3). The two glycosylation consensus sequences (N-X-S/T) in the ECD and the E/DRY/F sequence in the second ICL are highly conserved across invertebrate NPFRs and a sNPF/PrRP-type receptor (*A.rub*_sNPF/PrRP-R) in the starfish *Asterias rubens*. Two consensus protein kinase C (PKC) phosphorylation sequences (R/K-X-S/T) were observed in the C-terminal ICD of Hdh-NPFR as a similar feature with the consensus PKC and PKA phosphorylation sites (R-X-S/T or R-R/K-X-S/T) in the C-terminal ICD of *A.rub*_sNPF/PrRP-R, *Drosophila* NPFR (*D.mel*_NPFR), and human NPYR1/2. Comparison of the exon–intron structures and coding sequences (CDS) of *Hdh-NPFR* and *Hdh-NPFR-like* genes with those of *L.sta*_GPCR105, *A.rub*_sNPF/PrRP-R, and human and chicken *NPYR1/2/5* genes showed that *Hdh-NPFR* and *Hdh-NPFR-like* genes were more similar to *A.rub*_sNPF/PrRP-R and vertebrate *NPYR2* than to *NPYR1* and *NPYR5*, in terms of an intron insertion in the CDS in *NPYR1* and a longer third ICL in the CDS of *NPYR5* (Supplementary Figs. S3 and S4).

**Figure 2:**
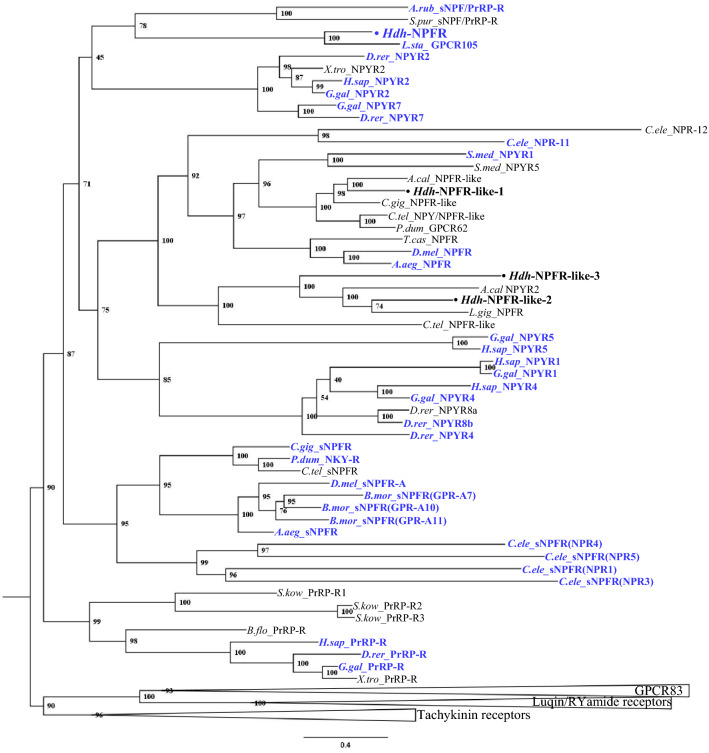
Phylogenetic tree analysis of Hdh-NPFR and three Hdh-NPFR-like receptors with bilaterian NPY/NPF-type, protostome sNPF-type, and deuterostome PrRP-type receptors. Luqin/RYamide-type, tachykinin-type, and GPCR83-type receptors were used as outgroups (condensed). Amino acid sequences of the receptors (Supplementary Table S2) were aligned and trimmed 270 residues were used to generate the maximum likelihood tree using W-IQ server. Ultrafast bootstrap values are given at each branch, and scale bar indicates amino acid substitutions per site. Deorphanized receptors for which receptor-ligand interactions have been experimentally characterized are colored in blue.

To investigate relationships of the Hdh-NPFR and Hdh-NPFR-like receptors with other bilaterian NPF/Y and related receptors, we performed a phylogenetic analysis using a sequence database with bilaterian NPF/NPY/sNPF receptors and other closely related receptors (tachykinin-, luqin-/RYamide-, PrRP-, and GPCR83-receptors as outgroups). This analysis revealed that the Hdh receptors were positioned in a large bilaterian NPF/Y receptor superfamily, with a bootstrap support of 71% (Fig. 2). More specifically, Hdh-NPFR was nested in the subclade composed of the deorphanized molluscan *L.sta*_NPFR, echinoderm sNPF/PrRP-Rs, and vertebrate NPYR2/7 with a relatively lower bootstrap value (45%); Hdh-NPFR-like-1 and Hdh-NPFR-like-2/-3 receptors were positioned in the subclades composed of lophotrochozoan and ecdysozoan NPF/Y receptors, and another lophotrochozoan NPFR-like receptors, respectively. All the Hdh-NPFR-like-1/2/3 showed a sister relationship with the subclade composed of vertebrate NPYR1/4/5/8.

### Subcellular localization of Hdh-NPFRs

Expression of hemagglutinin (HA)-tagged Hdh-NPFRs in human embryonic kidney 293 (HEK293) cells was verified by immunocytochemistry (ICC) with an anti-HA antibody. ICC analysis revealed that Hdh-NPFR and Hdh-NPFR-like receptors were mainly expressed in the cell membranes, although immunoreactive signals were also detected in the cytoplasm (Supplementary Fig. S5). The potency of Hdh-NPF to induce Hdh-NPFR internalization was determined by ICC. At 30 min post treatment of Hdh-NPF, Hdh-NPFR on the cell membrane moved into the cytoplasm, providing reliable evidence for the interaction of Hdh-NPF with Hdh-NPFR (Supplementary Fig. S6).

### Functional characterization of Hdh-NPFRs

**Figure 3:**
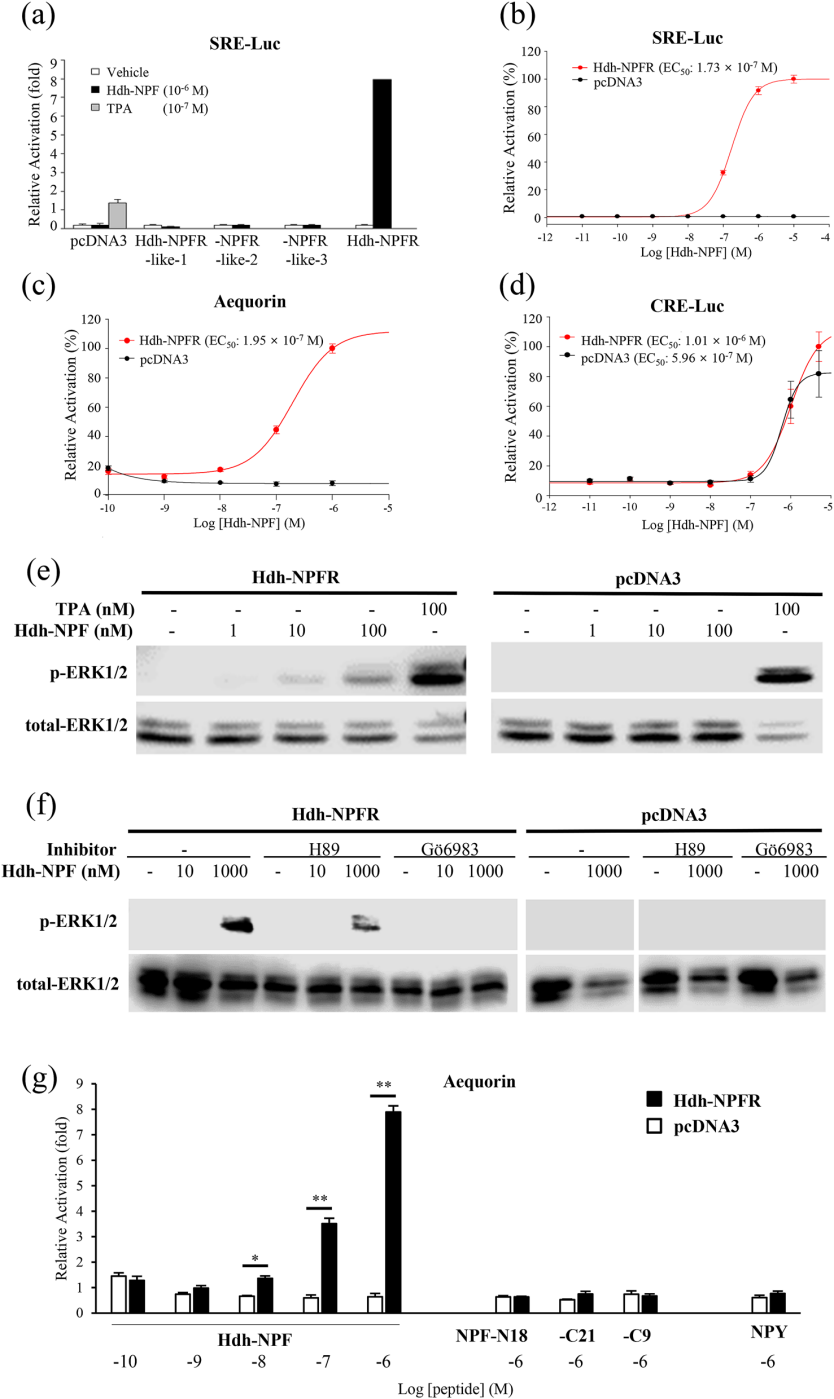
Hdh-NPF peptide induces the Hdh-NPFR signaling pathway. (**a**) SRE luciferase (SRE-Luc) reporter activities in Hdh-NPFR-related receptor-expressing HEK293 cells treated with Hdh-NPF. (**b**) Dose–response curve for SRE-Luc activities in Hdh-NPFR-expressing HEK293 cells treated with Hdh-NPF. Dose–response data are shown as percentages relative to the highest value (100% activation). (**c**) Dose–response curve for intracellular Ca^2+^ in Hdh-NPFR- and aequorin-expressing CHO-K1 cells treated with Hdh-NPF. (**d**) CRE-Luc reporter activities in Hdh-NPFR- or pcDNA3-transfected HEK293 cells. (**e**) Concentration dependence of Hdh-NPF-stimulated phosphorylation of ERK1/2 in Hdh-NPFR-expressing HEK293 cells. (**f**) Effects of the PKA inhibitor H89 and the PKC inhibitor Gö6983 on Hdh-NPFR-mediated activation of ERK1/2 in HEK293 cells. The cells were pre-incubated with or without the inhibitors (10^–5^ M) for 1 h and then stimulated with NPF. Full-length blots for phosphorylated and total ERK1/2 are presented in Supplementary Fig. S9. (**g**) Mean intracellular Ca^2+^ responses of Hdh-NPFR-transfected CHO-K1 cells by NPF, truncated Hdh-NPF-N18, -C21, -C9, and human NPY (see Table 1). Values for luciferase reporter and Ca^2+^ response assays are presented as the mean ± SEM (n = 3 or 4). Statistical significance was tested by Student’s t-test. *P < 0.05; **P < 0.01. TPA, 12-*O*-tetradecanoylphorbol-13-acetate; EC_50_, half-maximal effective concentration; H89, *N*-[2-(*p*-bromocinnamylamino)ethyl]-5-isoquinolinesulfonamide.

To further evaluate the signaling pathways involved in the abalone NPFR-related receptors, luciferase reporter systems under control of a minimal promoter containing a serum response element (SRE-Luc) or cAMP response element (CRE-Luc) were applied to determine ERK/MAPK activity, Ca^2+^ mobilization, and cAMP accumulation in Hdh-NPFRs-transfected HEK293 cells. Among the four Hdh-NPFR-related receptors, the synthesized Hdh-NPF peptide increased the SRE-Luc activity in the Hdh-NPFR-transfected HEK293 cells in a dose-dependent manner (Fig. 3a,b). Intracellular Ca^2+^ mobilization profiles of Hdh-NPFR were further investigated in Chinese hamster ovary (CHO)-K1 cells transfected with Hdh-NPFR- and aequorin-expressing plasmids with or without the promiscuous Gα15 plasmid. If Hdh-NPFR activation leads to Ca^2+^ increase without the promiscuous Gα15, this indicates that the Hdh-NPFR couples to the endogenous Gq protein to activate the phospholipase C (PLC)/inositol trisphosphate/PKC/Ca^2+^ release pathway in the CHO-K1 cells. Hdh-NPFR-transfected cells generated robust luminescence responses to Hdh-NPF in a dose-dependent manner, regardless of the presence of Gα15 (Fig. 3c and Supplementary Fig. S7a). However, the CRE-Luc-transfected HEK293 cells with or without Hdh-NPFR also responded to an increasing dose of Hdh-NPF, suggesting unknown receptor activity to heterologous Hdh-NPF in HEK293 cells (Fig. 3d). When we analyzed the CRE-Luc reporter system, human NPY inhibited forskolin-stimulated CRE-Luc activities in HEK293 cells expressing NPYR1/2 in a dose-dependent manner (Supplementary Fig. S8), indicating that NPY-mediated NPYR1/2 activation inhibits the cAMP/PKA signaling pathway, consistent with the findings of previous reports^39,41^.

Hdh-NPF treatments increased ERK phosphorylation in the Hdh-NPFR-transfected cells in a ligand-dependent manner (Fig. 3e). The Hdh-NPFR-mediated activation of ERK1/2 was abolished by the PKC inhibitor Gӧ6983 but not by the PKA inhibitor H89 (Fig. 3f), suggesting a fundamental importance for the Gq-mediated signaling pathway in the ERK1/2 MAPK cascade. In contrast, truncated Hdh-NPF peptides (NPF-N18, -C21, and -C9) and human NPY could not induce the Ca^2+^ mobilization response in the Hdh-NPFR-transfected cells (Fig. 3g). Two chimeric mixed peptides for Hdh-NPF and human NPY (NPF-Y and NPY-F) could not activate Ca^2+^ mobilization in the Hdh-NPFR-transfected cells (Table 1; Supplementary Fig. S7b).

**Table 1:** Amino acid sequences of peptides.

Peptide names	Sequences
Hdh-NPF	QDAMLAPPDRPSEFRSPDQLRQYLKALNEYYAIVGRPRF-NH_2_
Hdh-NPF-N18	QDAMLAPPDRPSEFRSPD-NH_2_
Hdh-NPF-C21	QLRQYLKALNEYYAIVGRPRF-NH_2_
Hdh-NPF-C9	YAIVGRPRF-NH_2_
NPF-Y	QDAMLAPPDRPSEFRSPDDMARYYSALRHYINLITRQRY-NH_2_
NPY-F	YPSKPDNPGEDAPAEQLRQYLKALNEYYAIVGRPRF-NH_2_
Human NPY	YPSKPDNPGEDAPAEDMARYYSALRHYINLITRQRY-NH_2_

### In silico model of the NPF-NPFR complex

**Figure 4:**
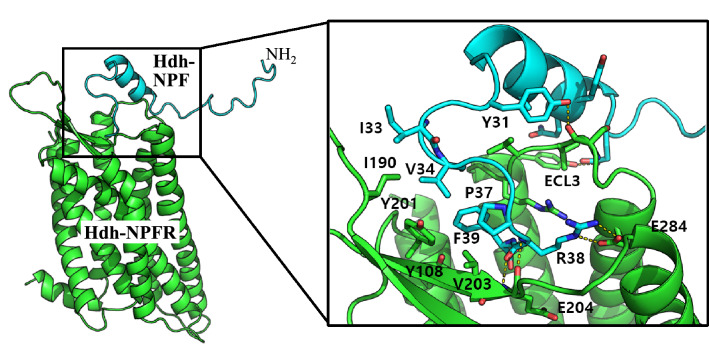
In silico docking model of the NPF-NPFR complex. The cartoon representation for Hdh-NPFR and Hdh-NPF is shown in green and cyan, respectively. The peptide binding interface (black box) is enlarged to show the details of the residue interactions, including R38 of Hdh-NPF and E284 in Hdh-NPFR. The helix region of Hdh-NPF (Q22–Y31) contacts the ECL3 region in Hdh-NPFR (S288–K293) connecting TMD6 and TMD7 outside the binding pocket.

Docking simulation was performed to predict the binding mode of Hdh-NPF peptide and estimate the critical interactions in the Hdh-NPFR binding pocket. The docking model suggested that the C-terminal sequence of Hdh-NPF peptide mainly interacts with Hdh-NPFR (Fig. 4). Specifically, R38 in abalone NPF forms a salt bridge with E204 and E284 in Hdh-NPFR, maintaining a stable hydrogen bond network. In addition, the I33, V34, and F39 residues in NPF have extensive hydrophobic interactions with Y108, I190, Y201, and V203 in Hdh-NPFR. Due to the small size of the binding pocket, only the C-terminal loop region penetrates the receptor and the rest of the peptide structure is exposed to the solvent. The internal peptide sequence near the helix region of Hdh-NPF (Q22–Y31) has many contact points to ECL3 in Hdh-NPFR (S288–K293), connecting TMD6 and TMD7 outside the binding pocket, which differs from the model of human NPY binding to Hdh-NPFR (Supplementary Fig. S10). This model suggests that the interaction between the specific residues of ECLs and Hdh-NPF peptide is critical for Hdh-NPFR activation.

### Tissue distribution of Hdh-NPF precursor and Hdh-NPFR transcripts

The expression patterns of *prepro-Hdh-NPF* and *Hdh-NPFR* transcripts were investigated in the cerebral ganglia (CG), pleuro-pedal ganglia (PPG), ovary, gills, intestine, and hepatopancreas of female abalone. The *prepro-Hdh-NPF* transcript was dominantly expressed in the CG, with a significantly higher level found in sexually mature females than in immature females (*P* < 0.05; Supplementary Fig. S11a). *Hdh-NPFR* transcripts were mainly expressed in the CG and PPG compared with the other tissues examined (Supplementary Fig. S11b). There were no significant differences of *Hdh-NPFR* transcript levels between immature and mature abalone tissues.

### Effects of Hdh-NPF on food consumption

**Figure 5:**
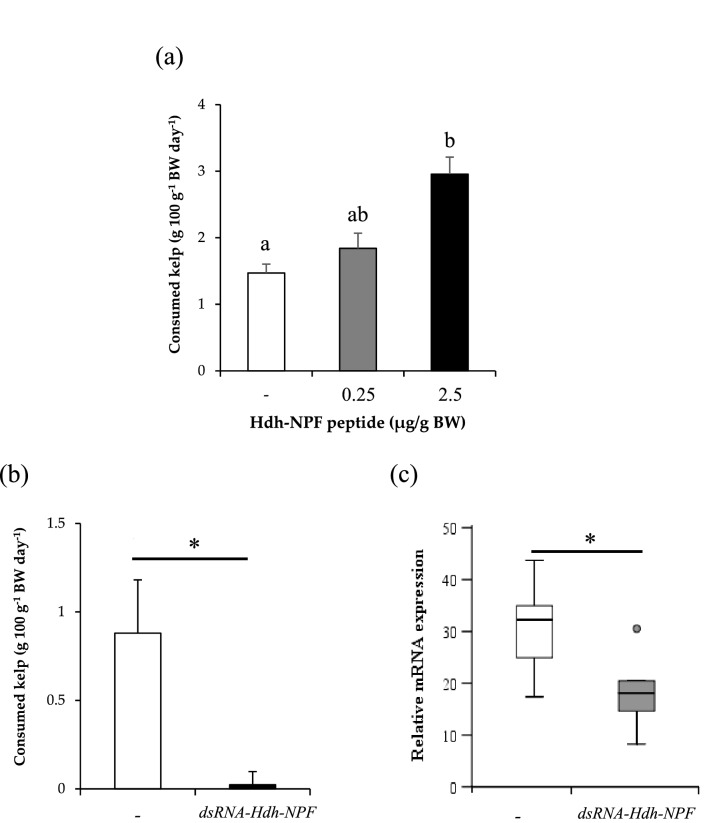
Effect of Hdh-NPF and double-stranded RNA of *Hdh-NPF* mRNA on food intake in abalone. (**a**) Effect of in vivo administration of Hdh-NPF peptide on kelp consumption in abalone. Each column and bar represent the mean and SEM (n = 11 per group), respectively. Significances of differences were evaluated by one-way analysis of variance with the Bonferroni correction method in comparison with the mollusk saline-injected group. Different lowercase letters indicate significant differences between treatments (*P* < 0.05). (**b**) Effects of *Hdh-NPF* knockdown on food intake. For RNA interference, individual abalone were injected with 165 μg of *dsRNA-Hdh-NPF* or the same volume of mollusk saline. Feeding assays were performed on the third day after injection. Results represent means ± SEM (n = 10 per group); *significant difference (*P* < 0.05) by Student’s *t*-test. (c) Relative *Hdh-NPF* transcript levels in the cerebral ganglia of abalone. The *Hdh-NPF* transcript levels in *dsRNA-Hdh-NPF*- or saline-injected abalone were measured by RT-qPCR. The ribosomal protein L-5 (*Hdh-RPL5*) was used as the internal control. Results represent means ± SEM (n = 10 per group); *statistical significance (*P* < 0.05) by Mann–Whitney *U* test.

Since NPF is known to be involved in energy metabolism in diverse invertebrate phyla^20^, we investigated whether Hdh-NPF affects food intake in Pacific abalone. Abalone injected with Hdh-NPF [2.5 μg/g body weight (BW)] showed a significant increase in food consumption (*P* < 0.05; Fig. 5a) at 24 h post-injection, whereas food consumption was significantly inhibited by injection of a double-stranded (ds) RNA for *Hdh-NPF* transcript (*dsRNA-Hdh-NPF*) compared with that observed following saline injection (*P* < 0.05; Fig. 5b). A significant decrease in *Hdh-NPF* transcript levels was also found in the *dsRNA-Hdh-NPF*-injected abalone group (*P* < 0.05; Fig. 5c), whereas no significant changes in *Hdh-GnRH* and *Hdh-APGWa* transcript levels were detected between saline- and *dsRNA-Hdh-NPF*-injected abalone groups (Supplementary Fig. S12).

## Discussion

In-depth screening of databases of diverse bilaterian genome and transcriptome sequences strongly suggested an ancient co-evolutionary history between NP precursor and receptor genes^42,43^. Elucidation of the endogenous ligands for orphan receptors in *D. melanogaster* and *C. elegans* has also offered important insight into the origin of NP signaling systems, dating back to the emergence of protostomes^44,45^. However, the NP systems of model species do not fully represent neofunctionalization and/or subfunctionalization of the ligand-receptor families of diverse animal phyla. Here, we present evidence that the abalone Hdh-NPF and Hdh-NPFR are evolutionally and functionally orthologous to the vertebrate NPY signaling system.

We identified two transcripts encoding a common Hdh-NPF precursor including the identical Hdh-NPF peptide sequence. Although a difference in a 26-nucleotides sequence at 3′-UTRs was found in the two *Hdh-NPF* transcripts, they were found to commonly encode a complete and identical open reading frame of Hdh-NPF precursor. The tandem *Hdh-NPF* genes were on the same scaffold in *Hdh* genome^40^, as seen in several invertebrate *NPF/Y* genes^4,46^. Given the conserved exon–intron structure and sequence similarities, it is likely that the two *Hdh-NPF* genes evolved from an initial gene duplication event at the chromosome level in Pacific abalone. A majority of NPF peptides have the evolutionally conserved C-terminal RxRF/Yamide sequences in diverse protostome invertebrates^19–21^. The molluscan NPF peptides including Hdh-NPF peptide comprised the conserved C-terminal RxRF/Y-amide and N-terminal PxxP residues, which are important for receptor affinity in vertebrate NPY signaling system^39^. In contrast, a phylogenetic analysis could not show a clear relationship between NPF/Y-related subfamily members, although the Hdh-NPF precursor was positioned in a clade of molluscan NPF group with a relatively high bootstrap value. This may be because of the variable signal peptide and the extensive divergence of C-terminal extension, resulting in the aa sequences to be of little or no use for large evolutionary distances. However, we determined that the exon–intron borders, especially the interposition of introns between the second and the third nucleotide of the second R codon in the C-terminal RxRF/Yamide motif, were highly conserved in the bilaterian *NPF/Y* genes and *FLP-34* gene, a *NPF/Y* ortholog in *C. elegans*^21^. These findings strongly suggest that the Hdh-NPF precursor is a genuine member of the invertebrate NPF group and that the bilaterian *NPF/NPY* genes originated from a common ancestor gene.

To date, *L.sta*_NPFR has been the only functionally verified receptor for molluscan NPF^20,30^. In this study, we identified the second molluscan NPFR from Pacific abalone that is characterized as a rhodopsin-like GPCR, with three ICDs and three ECD loops, an E/DRY/F sequence at the beginning region in the second IL, and seven TMDs^47,48^. The E/DRY/F sequence was highly conserved among the compared NPF/Y receptors, suggesting that the E/DRY/F motif plays a critical role in receptor function. For instance, mutation of the Arg residue in the E/DRY/F motif of human GPR40 (free fatty acid receptor 1), resulted in loss of agonist-induced functions, including Ca^2+^ mobilization, ERK activation, and receptor internalization^49^. PKC phosphorylation sites in the C-terminal ICD were similarly positioned in the Hdh-NPFR, *D.mel*_NPFR, *A.rub*_sNPF/PrRP-R, and human NPYR1/2, whereas consensus glycosylation sites were found within the predicted extracellular N-terminus of all the examined receptors. This suggests that the potential PKC phosphorylation sites in the ICD are not likely involved in the pivotal function of NPF/Y receptors, although the PKC-dependent cascade is clearly involved in activation of Hdh-NPFR as described in further detail below.

Through the sequence comparison and phylogenetic analysis, here we report that Hdh-NPFR-related receptors are belong to the NPF/Y receptor family distinct from the sNPFR and PrRP-R families in bilaterians. Consistent with this analysis, recent phylogenetic studies with novel NPF/NPY/PrRP/sNPF-related receptors in echinoderms and three lophotrochozoans (nemerteans, brachiopods, and phoronids) have revealed that all the examined molluscan NPFR-like sequences are orthologous to the vertebrate NPY receptors^22,43^. In addition, we suggest that Hdh-NPFR is most likely similar to vertebrate NPYR2 among the three vertebrate NPYR ancestors, the progenitors of the NPYR1, NPYR2, and NPYR5 subfamilies^4,50^. The first line of evidence is the sequence similarity between NPYR2 and Hdh-NPFR based on the BLAST and a phylogenetic analysis. Hdh-NPFR showed connections with the vertebrate NPYR2/7 cluster than with the NPYR1/4/5/8 cluster, although the bootstrap value of the branch was relatively low in this phylogenetic tree. In a previous report, the sequences of deorphanized *L.sta*_NPFR and *D.mel*_NPFR were closely related to the vertebrates NPYR2 receptor subtype in a phylogenetic tree based on Clustal W alignment and Jukes-Cantor distance analysis^51^. The second line of evidence is the exon–intron structure and the aa length of the third ICL of NPF/Y receptors. In human, chicken, and coelacanth fish (*Latimeria chalumnae*), a representative of a basal lineage of vertebrates, *NPYR1* show a conserved intron interposition in the nucleotide sequence encoding the 5th TMD region, whereas *NPYR2* and *NPYR5* have no intron sequences in the corresponding regions^52–56^. Similarly, *Hdh-NPFR* and *Hdh-NPFR-like* receptors had no interposition of introns in the entire open reading frame region. The third ICLs of vertebrate NPYR5 is at least four times longer than the corresponding region of other NPYR subtypes, which is clearly distinguishable from Hdh-NPFR and Hdh-NPFR-like receptors. Interestingly, comparison of the sequence of Hdh-NPFR with the starfish *A.rub*_sNPF/PrRP-R showed several common features, including the aforementioned predicted E/DRY/F motif in the second ICL, PKC phosphorylation sites in the C-terminal ICD and the exon–intron structure. However, functional studies on Hdh-NPFR and Hdh-NPFR-like receptors are needed for further characterization of molluscan *NPFR* gene duplication and the ancient complexity of the vertebrate NPY signaling system, since the *L.sta*_NPFR and *A.rub*_sNPF/PrRP-R were assumed to be vertebrate NPYR1 and PrRP-R homologs, respectively, based on pharmacological data^22,33^.

Since an earlier pioneering study demonstrated that Ls-NPF can activate the cognate receptor *L.sta*_NPFR in a model molluscan species, *L. stagnalis*^30^, the characterization of other functional NPF receptors and their downstream signaling pathways in mollusks has long been awaited. Here, we show that Hdh-NPFR is potently activated by the mature Hdh-NPF, and the ligand-activated receptor leads to activation of MAPK/ERK signaling through a Gq-PLC-PKC-dependent cascade and increase of intracellular Ca^2+^ mobilization, as is the case for *L.sta*_NPFR, human NPYR2, and chicken NPYR1/2/5/7 receptors^13,30,54,55^. This provisional pathway is supported by the results of western blot analyses showing that the PKA-specific inhibitor failed to inhibit the phosphorylation of ERK1/2, whereas the PKC inhibitor inhibited ERK1/2 phosphorylation in Hdh-NPFR-expressing cells after Hdh-NPF treatment. Nevertheless, we cannot rule out the possible inhibitory pathway of cAMP/PKA in the liganded Hdh-NPFR signal transduction system. In fact, *L.sta*_NPFR and vertebrate NPYRs are functionally coupled to more than one secondary messenger system^13,30,54,55^. The Hdh-NPFR along the cellular membrane translocated to an intracellular compartment in HEK293 cells at 30 min post treatment of Hdh-NPF, similar to NPY-stimulated human NPYR1^57^, suggesting that Hdh-NPFR transduces Ca^2+^ responses rapidly through the Gq-PLC-PKC signal cascade. Among the three vertebrate NPYR ancestors, NPYR1, NPYR2, and NPYR5, the most prominent pharmacological feature of NPYR2 is that it can bind N-terminal truncated peptide fragments^12,50^. In contrast, truncated Hdh-NPF peptides could not increase the intracellular Ca^2+^ mobilization in Hdh-NPFR-expressing cells, similar to the response of *L.sta*_NPFR-expressing cells treated with N-terminal truncated Ls-NPF^30^. These observations imply that the molluscan NPF signaling systems are not similar to those of vertebrate NPYR2, adding another level of complexity presented by the variation of bilateian NPF/Y system.

To identify the receptor residues that are important for Hdh-NPF binding, we performed a docking simulation to predict the binding mode of Hdh-NPF peptide and estimated the critical interactions in the Hdh-NPFR binding pocket. The docking model suggested that the C-terminal I33–F39 residues of Hdh-NPF are the major sites interacting with the binding pocket of Hdh-NPFR, under the reasonable assumption that R38 of Hdh-NPF interacts with E284 of TMD6 in the Hdh-NPFR extracellular region as the salt bridge formation between R35 and D287 in human NPYR1^39,58^. These data suggest that the ionic interaction contributed by the acidic E/D residue in TMD6 is critical for recognition of the ligands with a positive charge across bilaterian NPF/NPY receptors, including human NPYR2^59^. Similarly, the R33 residue in human NPY preferentially interacts with D292 (corresponding to E284 and D287 in Hdh-NPFR and NPYR1, respectively) in human NPYR2^39,60^. In this context, Hdh-NPFR would be a key and ancestral receptor for understanding the evolution of the NPY signaling system. In addition, we observed that the helix region of Hdh-NPF (Q22–Y31) contacts the ECL3 region of Hdh-NPFR (S288–K293) connecting TMD6 and TMD7 outside the binding pocket, which can explain the stronger energy of Hdh-NPF binding than that of human NPY binding to Hdh-NPFR. Taken together, the docking model presented plausible binding mode of Hdh-NPF with the conserved key interactions.

Since Stanley and Leibowitz^61^ revealed that injection of NPY into the hypothalamic paraventricular nucleus stimulates food intake in rats, the role of NPY/F signaling in regulation of feeding has been widely demonstrated in diverse vertebrate and invertebrate phyla^20^. In this study, a high dose of Hdh-NPF peptide injection could increase seaweed consumption, whereas injection of dsRNA for *Hdh-NPF* mRNA remarkably decreased food intake in Pacific abalone. These findings suggest that NPF production induces feeding behavior with a consequent increase of food intake, as observed across bilaterian species. In fact, feeding rhythms, ingestion rate, and digestive enzyme activities were positively correlated with the gene expression levels of *Hdh-NPF* in Pacific abalone^62^ and injection of NPF peptide was reported to significantly increase the filter feeding rate in the molluscan bivalve *R. philippinarum*^31^. In contrast, Ls-NPF showed no short-term effect on food intake in *L. stagnalis*^33,34^ and its role seems to be quite different from those of abalone NPF and mammalian NPY^9,10^. Instead, a significant increase in food intake started at 9 days after implantation of Ls-NPF^34^, suggesting that NPF is a long-term effector of food consumption in *L. stagnalis*. The possible roles of NPF in these regulatory mechanisms underlying food intake should be further determined in diverse molluscan species.

In conclusion, Hdh-NPF, Hdh-NPFR, and Hdh-NPFR-like receptors are eligible molecules to extend our understanding of the evolution and diversity of the metazoan NPF/Y-mediated signaling system. This work can contribute relevant information for practical applied in monitoring the physiological homeostasis and metabolism of Pacific abalone as an important economic species that is under threat due to extensive environmental stress from farming to meet the high market demand. Further elucidation of NPs with their cognate receptors in mollusks can provide opportunities to search endogenous NPs for uncharacterized human GPCRs, and to develop pharmaco-therapeutic ligands for human NP signaling systems.

## Materials and methods

### Nucleotide/amino acid sequence and phylogenetic analyses

Transcripts for the Hdh-NPF precursors (NCBI GenBank accession numbers MZ027150–MZ027151), Hdh-NPFR (MZ014382), and Hdh-NPFR-like receptor sequences (MZ014383–MZ014385) of Pacific abalone, *H. discus hannai*, were previously determined and were obtained from transcriptome databases^37,38^. Functional annotations were conducted by comparing the sequences against those in public databases, including the National Center for Biotechnology Information (NCBI) BLASTp program. Sequence alignments for bilaterian representative NPF/Y peptides (Supplementary Table S1) were performed using Clustal Omega Multiple Sequence Alignment with default parameters^63^. The software BOXSHADE (https://embnet.vital-it.ch/software/BOX_form.html) was used to highlight conserved amino acids. Sequence alignments for Hdh-NPFR-related receptor sequences were performed using CLC Genomics Workbench software. The prediction of transmembrane helices in Hdh-NPFR-related receptors was performed using the latest version of the TMHMM program^64^. The N-linked glycosylation and intracellular phosphorylation sites were predicted by the NetNGlyc and NetPhos servers, respectively (https://services.healthtech.dtu.dk/). The exon–intron boundaries were predicted by BLAST of NCBI Genome Workbench.

To generate phylogenetic trees, aa sequences of bilaterian NPF/Y and sNPF prepro-hormones (Supplementary Table S1), and NPF/Y, sNPF, and PrRP receptors (Supplementary Table S2) were retrieved from references^21,22^ and the NCBI nr repository. In total, 30 prepro-hormones and 83 receptors were aligned using MUSCLE in the online tool NGPhylogeny (iterative, 16 iterations, UPGMB as clustering method)^65^ and automatically trimmed using trimAL in the online tool NGPhylogeny^66^. The trimming contained a total of 39 and 270 residues for prepro-hormones and receptors, respectively, that were used to generate the maximum likelihood tree using W-IQ server v1.6.12^67^. The substitution models, PMB + I + G4 for prepro-hormones and LG + F + I + G4 for receptors, and the ultrafast bootstrap approximation approach and SH-aLRT 1000 replicates were used. Phylogenetic trees were visualized, using the free software package FigTree v1.4.3 by A. Rambaut at http://tree.bio.ed.ac.uk/software/figtree/.

### cDNA cloning and plasmid construction

Female Pacific abalone (9.2 cm shell length; 91.2 g BW) was purchased from a local dealer (Gangneung, Gangwon-do, Korea). Total RNA was extracted from the PPG using the RNeasy Mini kit (Qiagen, Valencia, CA, USA) and first-strand cDNA was synthesized using PrimeScript RT reagent kit with gDNA Eraser (Takara, Osaka, Japan) according to the manufacturer instructions. Polymerase chain reaction (PCR) was performed using the synthesized PPG cDNA as a template, PrimeSTAR HS DNA polymerase (Takara), and oligo primer sets (Table 2) for Hdh-NPFR-related receptors. The cycling conditions were as follows: 3 min at 98 °C; 35 cycles of 20 s at 98 °C, 20 s at 62 °C, and 1.5 min at 72 °C; and 5 min at 72 °C. The PCR-amplified products were digested by EcoRI and XbaI, and cloned into the restriction enzyme sites of the HA-tagged pcDNA3 expression vector (Invitrogen, Waltham, MA, USA). The plasmid constructs were analyzed to verify the correct sequence by Sanger sequencing.

**Table 2:** Oligo primer sequences used in the polymerase chain reaction.

Targets	Direction	Sequences (5′–3′)	Application
*Hdh-NPFR*	Sense	CGCGAATTCATGGATATGGAAGATATTCTGTTGA	cDNA cloning
*Hdh-NPFR*	Antisense	GCGTCTAGATTAGTATGGGGTGGTGTGTC	
*Hdh-NPFR-like-1*	Sense	CGCGAATTCATGGATGCCACCGTTGTAAGT	
*Hdh-NPFR-like-1*	Antisense	GCGTCTAGATTACGATTTCATTACGTTGAACTCCGA	
*Hdh-NPFR-like-2*	Sense	CGCGAATTCATGATCCAGAACTTCTTCACCAAC	
*Hdh-NPFR-like-2*	Antisense	GCGTCTAGATTAATTGCTCTGCCGGATCGA	
*Hdh-NPFR-like-3*	Sense	CGCGAATTCATGTTCCAAATGGCCAACCTG	
*Hdh-NPFR-like-3*	Antisense	GCGTCTAGATTATTCTAAACGTCGTTCATGAACAATGG	
*prepro-Hdh-NPF*	Sense	AATGGAAGTCACGTGTCAGG	RT-qPCR
*prepro-Hdh-NPF*	Antisense	GAGAGCCTTCAAGTACTGACG	
*Hdh-NPFR*	Sense	TATGGAACCCTTGGTTGCTC	
*Hdh-NPFR*	Antisense	TCGGTCTGCTTGATGTCTTG	
*Hdh-RPL5*	Sense	TCACCAACAAGGACATCATTTGTC	
*Hdh-RPL5*	Antisense	CAGGAGGAGTCCAGTGCAGTATG	
*prepro-Hdh-NPF*	Sense	TAATACGACTCACTATAGGGAGACTTTCTACCTGTTACTCAAAG	dsRNA amplification
*prepro-Hdh-NPF*	Antisense	TAATACGACTCACTATAGGGAGATACGCTCTGTCATCATAATG	
*prepro-Hdh-NPF*	Sense	AGTACAGTGTCATAACTCTTTCTCC	dsRNA injection followed by RT-qPCR verification
*prepro-Hdh-NPF*	Antisense	TCCCGACCTTTATTGGATCATTG	

Underlines and double-underlines indicate restriction enzyme recognition sites and T7 promoter sequence, respectively.

### Peptide synthesis

The abalone Hdh-NPF mature peptide sequence was predicted by SignalP-5.0 (http://www.cbs.dtu.dk/services-/SignalP) and NeuroPred (http://stagbeetle.animal.uiuc.edu/cgi-bin/neuropred.py) servers along with previous alignment data for NPF sequences^19^. Peptides for abalone Hdh-NPF and its truncated/mixed NPFs with NPY were synthesized by Anygen (Gwangju, Korea) with a purity of > 95% analyzed by high-performance liquid chromatography (Table 1).

### Luciferase reporter assay

HEK293 cells were cultured in Dulbecco’s modified Eagle medium (DMEM; Gibco, Loughborough, UK) containing 1% penicillin/streptomycin (P/S; Invitrogen) and 10% fetal bovine serum (FBS; Hyclone, GE Healthcare, Chicago, IL, USA). Sixteen hours before transfection, cultured HEK293 cells were seeded into 24-well plates (5 × 10^4^ cells/well). The cells were transfected with a luciferase reporter plasmid of CRE-Luc^68^ or SRE-Luc^69^ using a polyethyleneimine transfection reagent (Sigma-Aldrich, St. Louis, MO, USA), along with pcDNA3-HA plasmids containing Hdh-NPFR-related receptors, human NPYR1 and NPYR2 (kindly provided by Dr. Annette G. Beck-Sickinger, Leipzig University, Germany), and a pRSV-β-galactosidase expression plasmid as an internal control (100 ng of each plasmid/well) as previously reported^68^. After 3 h of transfection, the culture medium was replaced by new DMEM with 1% P/S and 10% FBS, and the HEK293 cells were further cultured for 30 h and then maintained in DMEM without FBS for 16 h. Finally, the HEK293 cells were treated with peptides, forskolin (Sigma-Aldrich), 12-*O*-tetradecanoylphorbol-13-acetate (TPA, Sigma-Aldrich), or the same volume of peptide-free medium as a vehicle for 6 h. The cells were lysed with a luciferase cell culture lysis buffer (Promega, Madison, WI, USA) and luciferase activities were analyzed using a microplate-luminometer (Berthold Tech., Bad Wildbad, Germany) and normalized by the β-galactosidase values detected by an absorbance microplate reader (Tecan, Mannedorf, Switzerland) at 405 nm.

### Ca^2+^ mobilization assay

CHO-K1 cells were maintained in DMEM-F12 Nutrient Mixture Ham supplemented with 10% FBS (Welgene) at 37 °C in 5% CO_2_. Cells grown in a monolayer to 60% confluency in 100 mm dishes (1.5 × 10^6^ cells) were transiently transfected with plasmids using FuGENE6 transfection reagent (Promega), according to the manufacturer’s recommendations. In brief, the transfection medium was prepared by combining 600 μl of DMEM-F12 with 2 μg of pcDNA3-HA-Hdh-NPFR plasmid together with 2 μg of a Ca^2+^ reporter aequorin plasmid with or without the promiscuous Gα15 expression plasmid (kind gifts from Dr. Young-Joon Kim, Gwangju Institute of Science and Technology, Gwangju, Korea) with 18 μl of FuGENE6 in a 5 ml polystyrene tube and incubated for 25 min at room temperature. The transfection mixture was added to CHO-K1 cells in 10 ml of fresh FBS-free medium and the cells were incubated at 37 °C in 5% CO_2_ overnight. On the day of the assay, the cells were detached from the culture dish by incubating with 3 ml of 5 mM EDTA in phosphate-buffered saline (PBS, pH 7.2; Thermo Fisher Scientific, Waltham, MA, USA) for 5 min. The cells were washed in 10 ml of phenol red-free DMEM/F-12 with 0.1% bovine serum albumin (BSA) and 1% P/S (DMEM-prf), and collected by brief centrifugation. Probenecid (1.25 mM final concentration, Invitrogen) and coelenterazine (5 μM, Gold Biotech., St. Louis, MO, USA) were added to resuspended CHO-K1 cells in 5 ml of DMEM-prf medium and the cells were gently agitated for 2.5 h using a magnetic stirrer at room temperature shielded from light. The cell suspension was diluted four times with DMEM-prf and incubated for a further 30 min. Just before the Ca^2+^ mobilization assay, peptide solutions were prepared in DMEM-prf and 50 μl aliquots were dispensed into 3 or 4 wells/peptide on 96-well microplates. DMEM-prf was used as a negative control. While stirring gently, 50 μl of the cell suspension was injected into a luminescence microplate reader (Berthold Tech.) using an automated injector unit. Luminescence was recorded for 20 s and for a further 10 s after injection of DMEM-prf including 0.2% Triton X-100 to measure the total Ca^2+^ response. The average luminescence from multiple replicate wells for a single concentration was calculated and normalized to the largest receptor response after subtraction of background values obtained from negative controls. Dose–response curves and EC_50_ values were obtained using Sigma Plot v.13 (Systat Software, Inc., San Jose, CA, USA).

### Immunocytochemistry

HEK293 cells were seeded on poly-d-lysine hydrobromide (Sigma-Aldrich)-coated coverslips, and pcDNA3-HA-Hdh-NPFR and related receptor constructs (300 ng each) were transfected as described for the luciferase reporter assays. After 30 h of culture, the cells were fixed with 4% paraformaldehyde in PBS (Wako, Osaka, Japan), and pretreated with PBS including 1% BSA and 0.1% Tween 20 (PBST buffer) at room temperature for 30 min. In the case of treatment with Hdh-NPF peptide, the cultured cells were further incubated for up to 30 min before fixation. The HEK293 cells were treated with a monoclonal HA primary antibody (1:5000 dilution; H9658, Sigma-Aldrich) in PBST at 4 °C for 16 h. After three washes with PBST, the cells were treated with a secondary antibody [anti-mouse IgG (H + L), F(ab′)2Fragment (AlexaFluor 488 Conjugate), 1:2000 dilution; Cell Signaling, Danvers, MA, USA] in PBST at room temperature for 1 h in a light-blocked chamber. After three washes with PBST, the cells were mounted with a mounting medium including DAPI (Abcam, Cambridge, UK), and were monitored with a fluorescence microscope (Eclipse E200; Nikon, Tokyo, Japan).

### Western blotting

HEK293 cells were seeded into 6-well plates (0.3 × 10^6^ cells/well) and transfected with 3 μg of pcDNA3-HA-Hdh-NPFR or pcDNA3 as described for the reporter assays. At 36 h post-transfection, the transfected cells were treated with Hdh-NPF (10^–9^–10^–7^ M), TPA (10^–7^ M), or the same volume of peptide-free medium as a vehicle for 10 min. After washing twice with PBS, the cells were lysed with RIPA buffer [150 mM NaCl, 1% Triton X-100, 0.5% sodium deoxycholate, 0.1% sodium dodecyl sulfate (SDS), 50 mM Tris–Cl, pH 8.0] including protease inhibitor cocktail and PhosSTOP (Roche, Basel, Switzerland) according to the manufacturer’s recommendation. To examine signaling pathways, Hdh-NPFR-transfected cells were pre-incubated with serum-free DMEM for 6 h at 37 °C and exposed to a PKA inhibitor (H89, 10^–5^ M; Sigma-Aldrich) or PKC inhibitor (Gӧ6983, 10^–5^ M; Sigma-Aldrich) for a further 60 min. After replacing the medium with fresh serum-free DMEM, the cells were exposed to Hdh-NPF peptide (10^–8^, 10^–6^ M) for 5 min at 37 °C. The cell lysates were separated by 10% SDS–polyacrylamide gel electrophoresis and subsequently transferred to nitrocellulose membranes (Pall, Ann Arbor, MI, USA). After blocking with TBST buffer (10 mM Tris–Cl,100 mM NaCl, 0.1% Tween-20, pH 7.5) containing 5% skim milk (BD Biosciences, Franklin Lakes, NJ, USA) at room temperature for 1 h, the membranes were incubated with a monoclonal mouse p44/42 MAPK or rabbit phospho-p44/42 MAPK (ERK1/2) (Thr202/Tyr204) primary antibody (1:2000 dilution; Cell Signaling) in TBST including 2.5% skim milk at 4 °C for 16 h. After three washes with TBST, the membranes were treated with goat anti-mouse IgG-HRP or goat anti-rabbit IgG-HRP secondary antibody (Santa Cruz Biotechnology, Santa Cruz, CA, USA) in TBST including 2.5% skim milk at room temperature for 2 h. The membranes were washed with TBST buffer twice and finally incubated with an enhanced chemiluminescence western blotting substrate reagent (Thermo Fisher Scientific) at room temperature for 5 min. The reactive bands were monitored using C-Digit 3600 Bolt Scanner (LI-COR, Lincoln, NE, USA).

### Construction of a receptor-ligand binding complex model

The three-dimensional (3D) structure of the 39-residue-long Hdh-NPF peptide was first modeled using PEP-FOLD3^70^, which predicts peptide structures from aa sequences with a de novo approach. The model structure that had the lowest energy potential according to OPEP (Optimized Potential for Efficient Structure Prediction) was considered as the final Hdh-NPF structure model. The 3D structure of Hdh-NPFR was modeled using SWISS-MODEL^71^. The best template for homology modeling, 5zbh (NPYR1 in T4 lysozyme), showed 31% sequence identity with Hdh-NPFR. The N- and C-terminal partial sequences were not modeled because none of the template structures covered the sequences and the N-terminal sequence in the ectodomain was disordered in the template structure.

The peptide and receptor model structures were used to perform docking simulation by RosettaScript^72^. The docking simulation consisted of an initial energy minimization, fast relax (all-atom refinement) application with distance constraints, and fast relax without a constraint. The distance constraint was defined between R35 in Hdh-NPF and E287 (6.59) in Hdh-NPFR given the results of recent structural studies on NPY and the cognate receptors^39,58,59^. The constraint ensured that the Hdh-NPF peptide is inserted into the binding pocket. The final relax application fully refined the complex structure without any constraints to check if the docked Hdh-NPF peptide structure is stably maintained in the binding pocket. Among 500 docking trials, the docking conformation with the lowest binding energy was considered as the final NPF-NPFR complex model (ΔΔG − 51.55 Rosetta Energy Unit by the REF2015 energy function).

### Real-time quantitative PCR (RT-qPCR)

The CG, PPG, ovary, gills, intestine, and hepatopancreas were dissected from adult female *H. discuss hannai* (n = 33; 8.7 ± 0.1 cm shell length; 73.9 ± 0.9 g BW) and immediately frozen in liquid nitrogen before storage at − 80 °C. The reproductive stage of abalone gonads was classified according to a previous study^58^. Total RNA was extracted using the RNeasy Mini kit (Qiagen) and 1 μg of RNA was reverse-transcribed to first-strand cDNA using the PrimeScript RT reagent kit (Takara). The ribosomal protein L-5 gene (*Hdh-RPL5*) was used as an internal reference for RT-qPCR as previously described^73^. Gene-specific primers for amplifying the *Hdh-NPF* precursor and *Hdh-NPFR* genes are listed in Table 2.

### Effect of Hdh-NPF on food intake

Pacific abalone (33.28 ± 0.81 g BW) were maintained in a flow-through seawater aquarium (18 ± 1 °C; 400 L) for 2 weeks, and fed ad libitum on kelp (*Saccharina japonica*) before use in experiments. To reduce consumption rate bias based on individual variation, the abalone were starved for 48 h, placed in individual containers, and refed for 24 h before the experiment. On the day of the experiment, kelp pieces were divided into two equal parts, blotted, and weighed to obtain the wet mass (g) before and after the feeding period. To correct for autogenic changes in kelp mass over time, one part was secured to the rim of the treatment cage and the other was secured to the control cage without abalone. Abalone were weighed prior to each assay (n = 11 per group) and 350 μl of mollusk saline (13 g HEPES, 25.66 g NaCl, 0.82 g KCl, 1.69 g CaCl_2_, 10.17 g MgCl_2_, 2.56 g Na_2_SO_4_, 1.0 L dH_2_O; pH 7.2) including Hdh-NPF (0.25 or 2.5 μg/g BW) was injected into the adduct muscle sinus using a 26-gauge needle. Control abalone were injected with the same volume of mollusk saline. Injected abalone were individually placed in a cage (15.5 × 11 × 6.5 cm) with flow-through seawater and supplied with seawater-immersed kelp equivalent to 7% of the BW. Food intake was assessed at 24 h post-injection as follows: Consumption (W) = [Wi × (WCf/WCi) − Wf], where Wi is the initial wet kelp weight, Wf is the remaining wet kelp weight, and WC is an autogenic control to determine the permeation of water into kelp during the feeding time. Consumption values were standardized for abalone BW to 100 g.

### dsRNA of Hdh-NPF (*dsRNA-Hdh-NPF*)

A 412-bp cDNA fragment (nucleotides 48–460) of Hdh-NPF precursor (NCBI accession numbers MZ027150–MZ027151) was amplified by PCR using gene-specific primers with a T7 promoter tag (Table 2) and a CG template. The PCR fragment was sub-cloned into the pGEM-T Easy vector (Promega) and the insert was analyzed to evaluate the sequence integrity. A second round of PCR was performed with the same T7-tagged primers and the *Hdh-NPF* amplicons as templates for the synthesis of *dsRNA-Hdh-NPF* using the T7 Ribomax Express RNAi system (Promega). As described in the preceding section, Pacific abalone (28.56 ± 0.61 g BW; n = 20) were starved, refed, and injected with 120 μl of 1.38 μg/μl *dsRNA-Hdh-NPF* or the same volume of mollusk saline into the adduct muscle sinus (n = 10 per group). The quantity of 165 μg of *dsRNA-Hdh-NPF*, corresponding to a mean concentration of 10 μg of dsRNA per gram of abalone BW without shell weight, was within the range of dsRNA quantities injected into other invertebrates (4–8 μg for shrimp *Litopenaeus vannamei* and 20 μg for oyster *Crassostrea gigas* per gram BW)^74,75^. At 72 h post-injection, abalone were supplied with seawater-immersed kelp and food consumption was measured for 24 h. Immediately after the feeding assay, CG tissues were dissected from abalone, frozen in liquid nitrogen, and then stored at − 80 °C until RNA extraction. Total RNA extraction, cDNA synthesis, and RT-qPCR for *Hdh-NPF*, *Hdh-GnRH*, and *Hdh-APGWa* transcripts were conducted as described above, except for the primers used (Table 2)^73,76^.

## Supplementary Information

Supplementary Information 1.

Supplementary Information 2.

Supplementary Information 3.

Supplementary Information 4.

## Data Availability

The sequences generated in this study have been deposited in the NCBI GenBank database (accession numbers MZ027150–MZ027151 for Hdh-NPF precursors, MZ014382 for Hdh-NPFR, and MZ014383–MZ014385 for Hdh-NPFR-like receptors).

## References

[1] Taghert PH, Nitabach MN (2012). Peptide neuromodulation in invertebrate model systems. Neuron.

[2] van den Pol AN (2012). Neuropeptide transmission in brain circuits. Neuron.

[3] Marder E (2012). Neuromodulation of neuronal circuits: Back to the future. Neuron.

[4] Elphick MR, Mirabeau O, Larhammar D (2018). Evolution of neuropeptide signalling systems. J. Exp. Biol..

[5] Jékely G (2013). Global view of the evolution and diversity of metazoan neuropeptide signaling. Proc. Natl. Acad. Sci. U.S.A..

[6] Zandawala M, Tian S, Elphick MR (2018). The evolution and nomenclature of GnRH-type and corazonin-type neuropeptide signaling systems. Gen. Comp. Endocrinol..

[7] Tatemoto K, Carlquist M, Mutt V (1982). Neuropeptide Y–a novel brain peptide with structural similarities to peptide YY and pancreatic polypeptide. Nature.

[8] Holzer P, Reichmann F, Farzi A (2012). Neuropeptide Y, peptide YY and pancreatic polypeptide in the gut-brain axis. Neuropeptides.

[9] Clark JT, Kalra PS, Crowley WR, Kalra SP (1984). Neuropeptide Y and human pancreatic polypeptide stimulate feeding behavior in rats. Endocrinology.

[10] Loh K, Herzog H, Shi YC (2015). Regulation of energy homeostasis by the NPY system. Trends Endocrinol. Metab..

[11] Reichmann F, Holzer P (2016). Neuropeptide Y: A stressful review. Neuropeptides.

[12] Michel MC (1998). XVI. International Union of Pharmacology recommendations for the nomenclature of neuropeptide Y, peptide YY, and pancreatic polypeptide receptors. Pharmacol. Rev..

[13] Rose PM (1995). Cloning and functional expression of a cDNA encoding a human type 2 neuropeptide Y receptor. J. Biol. Chem..

[14] Larhammar D (1992). Cloning and functional expression of a human neuropeptide Y/peptide YY receptor of the Y1 type. J. Biol. Chem..

[15] Daniels AJ (1995). Structure-activity relationship of novel pentapeptide neuropeptide Y receptor antagonists is consistent with a noncontinuous epitope for ligand-receptor binding. Mol. Pharmacol..

[16] Lindner D, Stichel J, Beck-Sickinger AG (2008). Molecular recognition of the NPY hormone family by their receptors. Nutrition.

[17] Brothers SP, Wahlestedt C (2010). Therapeutic potential of neuropeptide Y (NPY) receptor ligands. EMBO Mol. Med..

[18] Yi M (2018). A promising therapeutic target for metabolic diseases: neuropeptide Y receptors in humans. Cell. Physiol. Biochem..

[19] Nässel DR, Wegener C (2011). A comparative review of short and long neuropeptide F signaling in invertebrates: Any similarities to vertebrate neuropeptide Y signaling?. Peptides.

[20] Fadda M (2019). Regulation of feeding and metabolism by neuropeptide F and short neuropeptide F in invertebrates. Front. Endocrinol. (Lausanne).

[21] Fadda M (2020). NPY/NPF-related neuropeptide FLP-34 signals from serotonergic neurons to modulate aversive olfactory learning in *Caenorhabditis elegans*. J. Neurosci..

[22] Yañez-Guerra LA (2020). Echinoderms provide missing link in the evolution of PrRP/sNPF-type neuropeptide signalling. Elife.

[23] Maule AG (1991). Neuropeptide F: A novel parasitic flatworm regulatory peptide from *Moniezia expansa* (Cestoda: Cyclophyllidea). Parasitology.

[24] Chung BY (2017). Drosophila neuropeptide F signaling independently regulates feeding and sleep-wake behavior. Cell Rep..

[25] van Wielendaele P, Dillen S, Zels S, Badisco L, Vanden Broeck J (2013). Regulation of feeding by Neuropeptide F in the desert locust, *Schistocerca gregaria*. Insect Biochem. Mol. Biol..

[26] Sedra L, Lange AB (2016). Cloning and expression of long neuropeptide F and the role of FMRFamide-like peptides in regulating egg production in the Chagas vector, *Rhodnius prolixus*. Peptides.

[27] Ameku T (2018). Midgut-derived neuropeptide F controls germline stem cell proliferation in a mating-dependent manner. PLoS Biol..

[28] Liu W (2019). Neuropeptide F regulates courtship in *Drosophila* through a male-specific neuronal circuit. Elife.

[29] Rosenberg G (2014). A new critical estimate of named species-level diversity of the recent Mollusca. Am. Malacol. Bull..

[30] Tensen CP (1998). Molecular cloning and characterization of an invertebrate homologue of a neuropeptide Y receptor. Eur. J. Neurosci..

[31] Wang X, Miao J, Liu P, Pan L (2017). Role of neuropeptide F in regulating filter feeding of Manila clam, *Ruditapes philippinarum*. Comp. Biochem. Physiol. B Biochem. Mol. Biol..

[32] Jing J (2007). From hunger to satiety: Reconfiguration of a feeding network by *Aplysia* neuropeptide Y. J. Neurosci..

[33] de Jong-Brink M, ter Maat A, Tensen CP (2001). NPY in invertebrates: Molecular answers to altered functions during evolution. Peptides.

[34] de Jong-Brink M, Reid CN, Tensen CP, Ter Maat A (1999). Parasites flicking the NPY gene on the host's switchboard: Why NPY?. FASEB J..

[35] Cook PA (2019). Worldwide abalone production statistics. J. Shellfish Res..

[36] Morash AJ, Alter K (2016). Effects of environmental and farm stress on abalone physiology: Perspectives for abalone aquaculture in the face of global climate change. Rev. Aquac..

[37] Kim MA (2017). Alternative splicing profile and sex-preferential gene expression in the female and male Pacific Abalone *Haliotis discus hannai*. Genes (Basel).

[38] Kim MA (2019). Neural ganglia transcriptome and peptidome associated with sexual maturation in female Pacific abalone (*Haliotis discus hannai*). Genes (Basel).

[39] Pedragosa-Badia X, Stichel J, Beck-Sickinger AG (2013). Neuropeptide Y receptors: how to get subtype selectivity. Front. Endocrinol. (Lausanne).

[40] Nam BH (2017). Genome sequence of pacific abalone (*Haliotis discus hannai*): the first draft genome in family Haliotidae. Gigascience.

[41] Cabrele C, Beck-Sickinger AG (2000). Molecular characterization of the ligand-receptor interaction of the neuropeptide Y family. J. Pept. Sci..

[42] Mirabeau O, Joly JS (2013). Molecular evolution of peptidergic signaling systems in bilaterians. Proc. Natl. Acad. Sci. U.S.A..

[43] Thiel D, Yañez-Guerra LA, Franz-Wachtel M, Hejnol A, Jékely G (2021). Nemertean, brachiopod and phoronid neuropeptidomics reveals ancestral spiralian signalling systems. Mol. Biol. Evol..

[44] Hewes RS, Taghert PH (2001). Neuropeptides and neuropeptide receptors in the *Drosophila melanogaster* genome. Genome Res..

[45] Frooninckx L (2012). Neuropeptide GPCRs in *C. elegans*. Front. Endocrinol. (Lausanne).

[46] Veenstra JA (2011). Neuropeptide evolution: neurohormones and neuropeptides predicted from the genomes of *Capitella teleta* and *Helobdella robusta*. Gen. Comp. Endocrinol..

[47] Ballesteros J (1998). Functional microdomains in G-protein-coupled receptors. The conserved arginine-cage motif in the gonadotropin-releasing hormone receptor. J. Biol. Chem..

[48] Pearce LR, Komander D, Alessi DR (2010). The nuts and bolts of AGC protein kinases. Nat. Rev. Mol. Cell Biol..

[49] Guo S, Zhang J, Zhang S, Li J (2015). A single amino acid mutation (R104P) in the E/DRY motif of GPR40 impairs receptor function. PLoS ONE.

[50] Larhammar D, Salaneck E (2004). Molecular evolution of NPY receptor subtypes. Neuropeptides.

[51] Garczynski SF, Brown MR, Shen P, Murray TF, Crim JW (2002). Characterization of a functional neuropeptide F receptor from *Drosophila melanogaster*. Peptides.

[52] Ammar DA (1996). Characterization of the human type 2 neuropeptide Y receptor gene (NPY2R) and localization to the chromosome 4q region containing the type 1 neuropeptide Y receptor gene. Genomics.

[53] Herzog H (1997). Overlapping gene structure of the human neuropeptide Y receptor subtypes Y1 and Y5 suggests coordinate transcriptional regulation. Genomics.

[54] He C (2016). Molecular characterization of three NPY receptors (Y2, Y5 and Y7) in chickens: Gene structure, tissue expression, promoter identification, and functional analysis. Gen. Comp. Endocrinol..

[55] Gao S (2017). Molecular characterization of neuropeptide Y (NPY) receptors (Y1, Y4 and Y6) and investigation of the tissue expression of their ligands (NPY, PYY and PP) in chickens. Gen. Comp. Endocrinol..

[56] Larhammar D, Bergqvist CA (2013). Ancient grandeur of the vertebrate neuropeptide Y system shown by the coelacanth *Latimeria chalumnae*. Front. Neurosci..

[57] Gicquiaux H (2002). Rapid internalization and recycling of the human neuropeptide Y Y(1) receptor. J. Biol. Chem..

[58] Yang Z (2018). Structural basis of ligand binding modes at the neuropeptide Y Y(1) receptor. Nature.

[59] Tang T (2021). Structural basis for ligand recognition of the neuropeptide Y Y(2) receptor. Nat. Commun..

[60] Merten N (2007). Receptor subtype-specific docking of Asp6.59 with C-terminal arginine residues in Y receptor ligands. J. Biol. Chem..

[61] Stanley BG, Leibowitz SF (1984). Neuropeptide Y: Stimulation of feeding and drinking by injection into the paraventricular nucleus. Life Sci..

[62] Gao X, Pang G, Luo X, You W, Ke C (2021). Effects of light cycle on circadian feeding activity and digestive physiology in *Haliotis discus hannai*. Aquaculture.

[63] Madeira F (2019). The EMBL-EBI search and sequence analysis tools APIs in 2019. Nucleic Acids Res..

[64] Krogh A, Larsson B, von Heijne G, Sonnhammer EL (2001). Predicting transmembrane protein topology with a hidden Markov model: Application to complete genomes. J. Mol. Biol..

[65] Lemoine F (2019). NGPhylogeny.fr: new generation phylogenetic services for non-specialists. Nucleic Acids Res..

[66] Capella-Gutiérrez S (2009). trimAl: A tool for automated alignment trimming in large-scale phylogenetic analyses. Bioinformatics.

[67] Trifinopoulos J (2016). W-IQ-TREE: A fast online phylogenetic tool for maximum likelihood analysis. Nucleic Acids Res..

[68] Ko H, Park W, Kim DJ, Kobayashi M, Sohn YC (2007). Biological activities of recombinant Manchurian trout FSH and LH: Their receptor specificity, steroidogenic and vitellogenic potencies. J. Mol. Endocrinol..

[69] Oh DY (2005). Membrane-proximal region of the carboxyl terminus of the gonadotropin-releasing hormone receptor (GnRHR) confers differential signal transduction between mammalian and nonmammalian GnRHRs. Mol. Endocrinol..

[70] Shen Y, Maupetit J, Derreumaux P, Tufféry P (2014). Improved PEP-FOLD approach for peptide and miniprotein structure prediction. J. Chem. Theory. Comput..

[71] Waterhouse A (2018). SWISS-MODEL: Homology modelling of protein structures and complexes. Nucleic Acids Res..

[72] Fleishman SJ (2011). RosettaScripts: a scripting language interface to the Rosetta macromolecular modeling suite. PLoS ONE.

[73] Kim TH (2017). Characterization and spatiotemporal expression of gonadotropin-releasing hormone in the Pacific abalone, *Haliotis discus hannai*. Comp. Biochem. Physiol. A Mol. Integr. Physiol..

[74] Robalino J (2004). Induction of antiviral immunity by double-stranded RNA in a marine invertebrate. J. Virol..

[75] Fabioux C, Corporeau C, Quillien V, Favrel P, Huvet A (2009). *In vivo* RNA interference in oyster–vasa silencing inhibits germ cell development. FEBS J..

[76] Kim KS, Kim TH, Kim MA, Lee JS, Sohn YC (2018). Expression profile and reproductive regulation of APGWamide in Pacific abalone (*Haliotis discus hannai*). Comp. Biochem. Physiol. A Mol. Integr. Physiol..

