# Supplementary material for: NPF activates a specific NPF receptor and regulates food intake in Pacific abalone Haliotis discus hannai
Source: Sci Rep. 2021 Oct 22;11:20912. doi: 10.1038/s41598-021-00238-1 (PMC8536682; doi:10.1038/s41598-021-00238-1)
Supplement: Supplementary file 1 — Supplementary Information 1. [file 41598_2021_238_MOESM1_ESM.docx]

Supplementary Material for

**NPF activates a specific NPF receptor and regulates food intake in Pacific abalone *Haliotis discus hannai***

Kyeong Seop Kim^1^, Mi Ae Kim^1,2^, Keunwan Park^3^ & Young Chang Sohn^1,*^

^1^Department of Marine Molecular Biosciences, Gangneung-Wonju National University, 7 Jukheon-gil, Gangneung, Gangwon 25457, Republic of Korea

^2^East Coast Life Sciences Institute, Gangneung-Wonju National University, 7 Jukheon-gil, Gangneung, Gangwon 25457, Republic of Korea

^3^Natural Product Informatics Research Center, KIST Gangneung Institute of Natural Products, Gangneung, Gangwon 25451, Republic of Korea

*Correspondence and requests for materials should be addressed to Y.C.S. (email: [ycsohn@gwnu.ac.kr](mailto:ycsohn@gwnu.ac.kr))

**
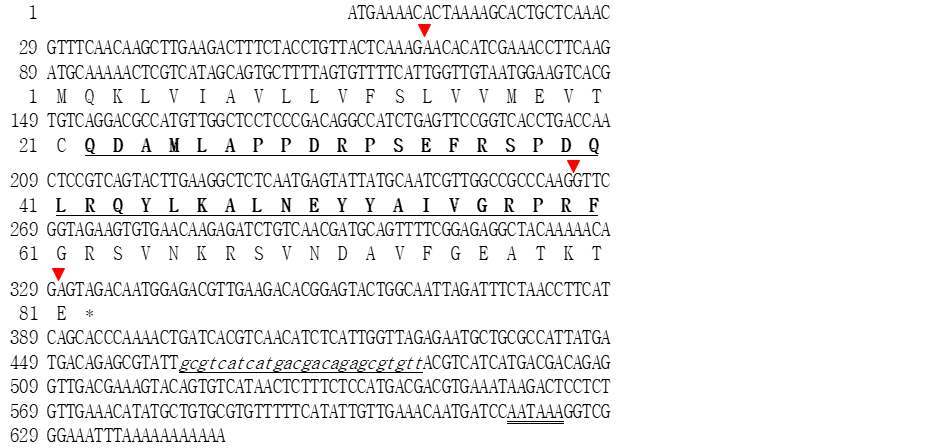
**


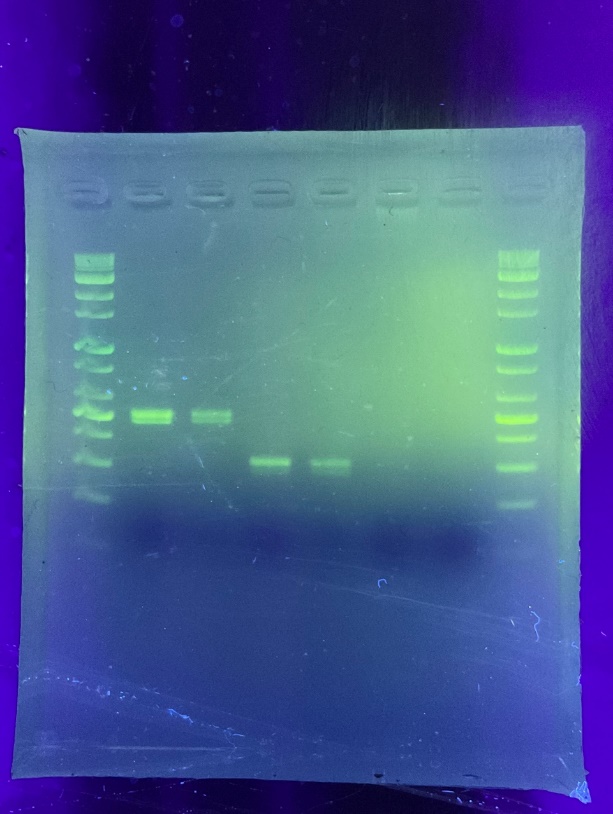


1 2 3 4 5 6 7 8

600

800

1000

500

400

300

200

bp

# **Fig. S1.** cDNA and deduced amino acid sequences of *H. discus hannai* NPF (Hdh-NPF) precursors. Two *prepro-Hdh-NPF* transcripts had identical sequences, except for the 26-nucleotides insertion (underlined lowercase-italic letters) in a variant of *Hdh-NPF* precursor. Mature Hdh-NPF amino acid residues are shown in underlined bold letters. The stop codon and polyadenylation signal are denoted by an asterisk and a double underline, respectively. Red triangles indicate the interposition site of introns. The sequences generated in this study have been deposited in the NCBI GenBank database (Accession Nos. MZ027150-MZ027151). Results of agarose gel electrophoresis show two reverse-transcribed PCR bands from the total RNAs of the CG (lanes 2, 4) and PPG (lanes 3, 5). Distilled water additions (lanes 6, 7) did not show amplicons in the PCR. An oligo primer set of 5ʹ-AATGGAAGTCACGTGTCAGG-3ʹ and 5ʹ-TCCCGACCTTTATTGGATCATTG-3ʹ for 2, 3, 6 lanes; 5ʹ-GACCAACTCCGTCAGTACTTG-3ʹ and 5ʹ-GTCAACCTCTGTCGTCATGATG-3ʹ for 4, 5, 7 lanes. Lanes 1, 8: 100-bp molecular weight marker.

**
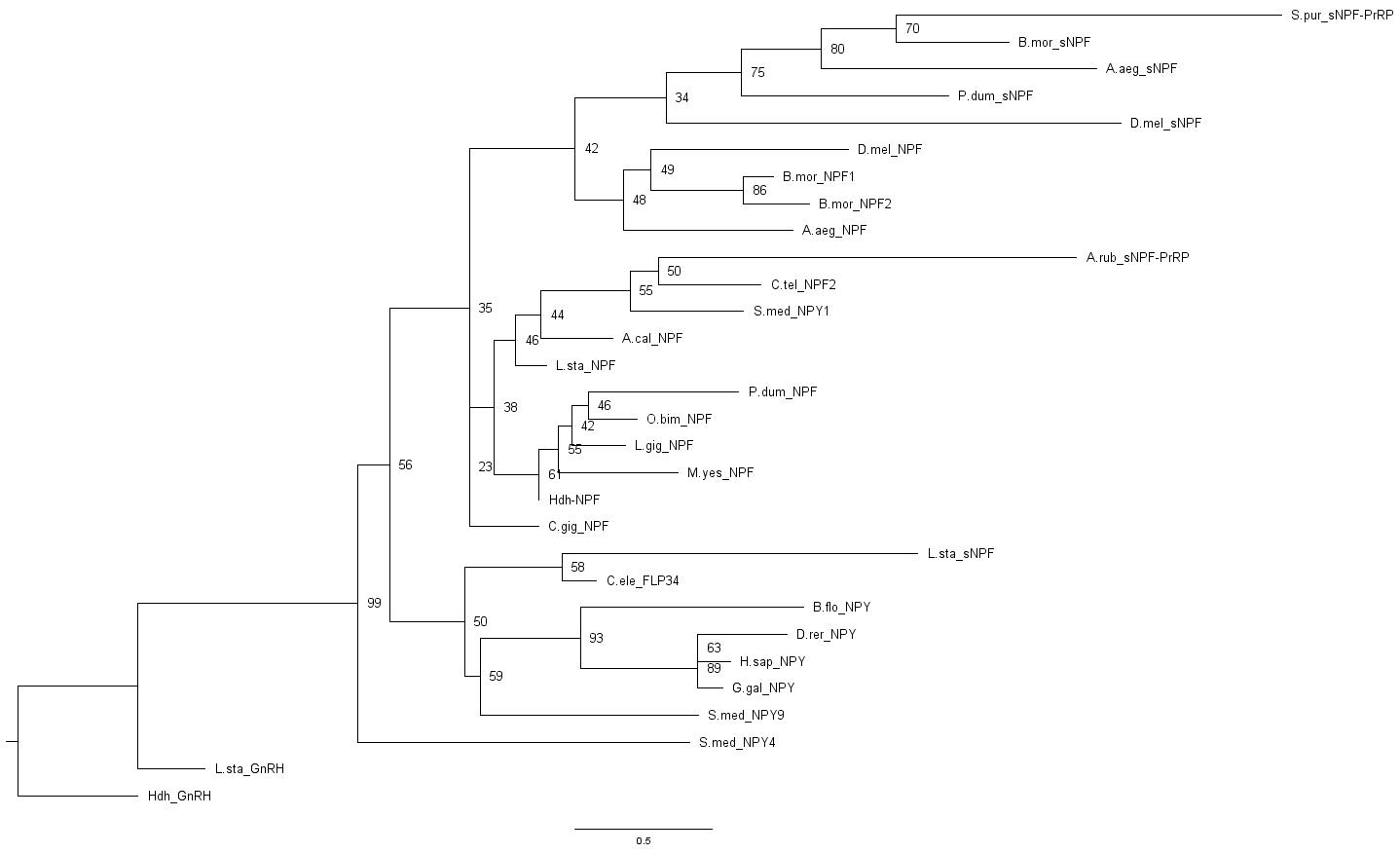
**

**Fig. S2.** Phylogenetic tree analysis based on amino acid sequences of NPF/Y- and sNPF-related precursors. Hdh-GnRH and *L.sta*_GnRH precursors were used as an outgroup. The trimmed amino acid sequences were used for each neuropeptide precursor (given in Supplementary Table S1) and maximum likelihood tree was generated using W-IQ server v1.6.12. Bootstrap values are given at each branch. The scale bar indicates amino acid substitutions per site.

**
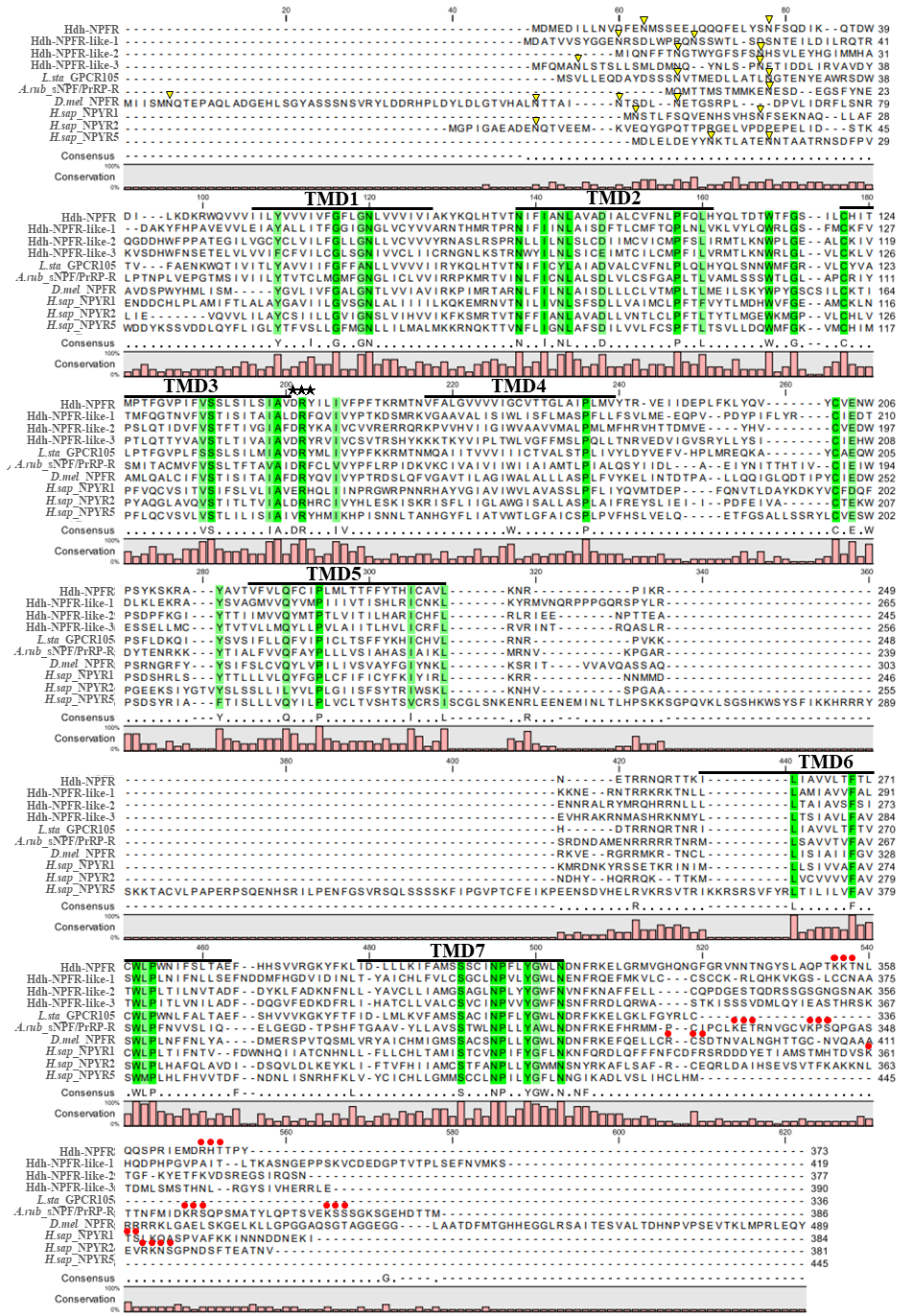
**

**Fig. S3.** Amino acid sequence alignment of Hdh-NPFR-related receptors. The predicted seven transmembrane domains (TMD1–7) are indicated above the alignment. Dark and light green shaded amino acids represent residues with >90% and >80% conserved regions, respectively. Potential N-linked glycosylation sites, the characteristic E/DRY/F sequence of rhodopsin-like GPCR, and consensus PKC and PKA phosphorylation sites are denoted with yellow arrowheads, black stars, and red dots on the amino acids, respectively. Sequence abbreviations are listed in Supplementary Table S2.

**
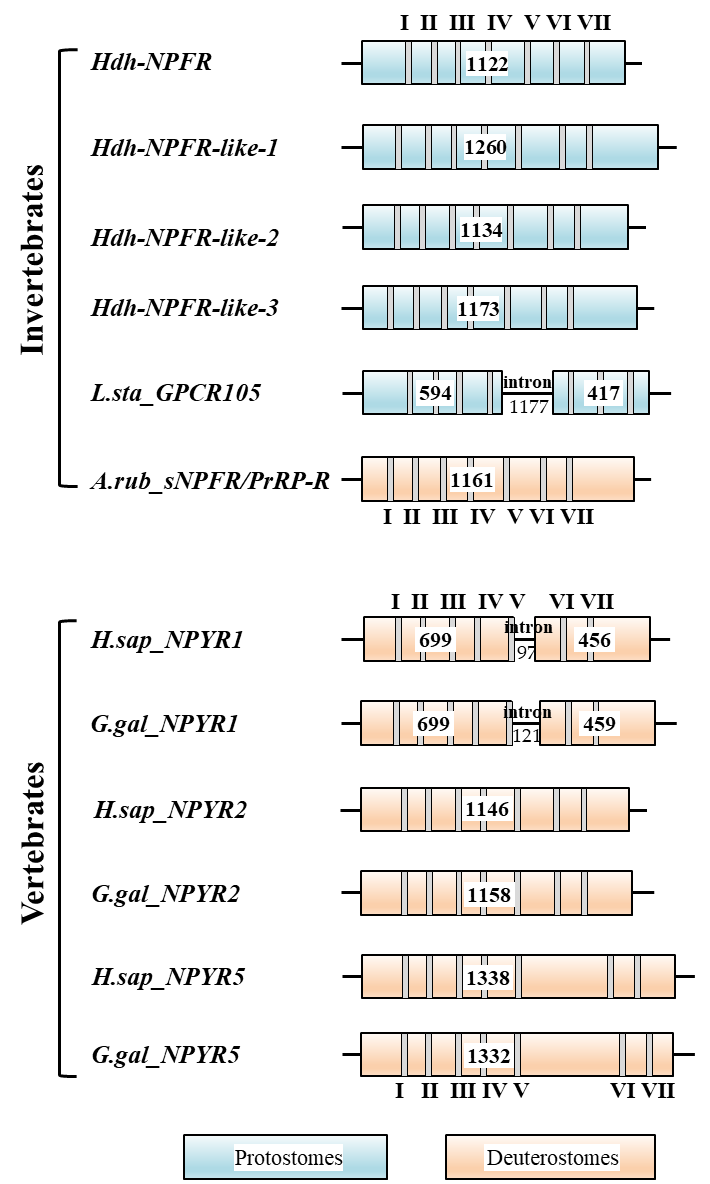
**

**Fig. S4.** Schematic architecture of invertebrate *Hdh-NPFR*-related receptors, and vertebrate *NPYR1*, *NPYR2*, and *NPYR5* genes. Exons and introns are represented by boxes and lines, respectively, and numbers in the boxes and under the lines indicate the nucleotide lengths (bp). Roman numbers (Ⅰ - Ⅶ) indicate the regions that code for the transmembrane regions (shown as vertical grey bars). Numbers under the lines indicate the nucleotide lengths of introns. Gene accession numbers in the NCBI databases are as follows: HDIH16787 for Hdh-NPFR; HDIH08377 for Hdh-NPFR-like-1; HDIH04188 for Hdh-NPFR-like-2; HDIH22022 for Hdh-NPFR-like-3; FCFB01039178.1 and FCFB01039178.1 for *L.sta_GPCR105*; NC_047070.1 for *A.rub_sNPF/PrRP-R*; NC_000004.12 for *H.sap_NPYR1*; NC_000004.11 for *H.sap_NPYR2/5*; NC_052535.1 for *G.gal_NPYR1/2/5.* Sequence abbreviations are listed in Supplementary Table S2.

**
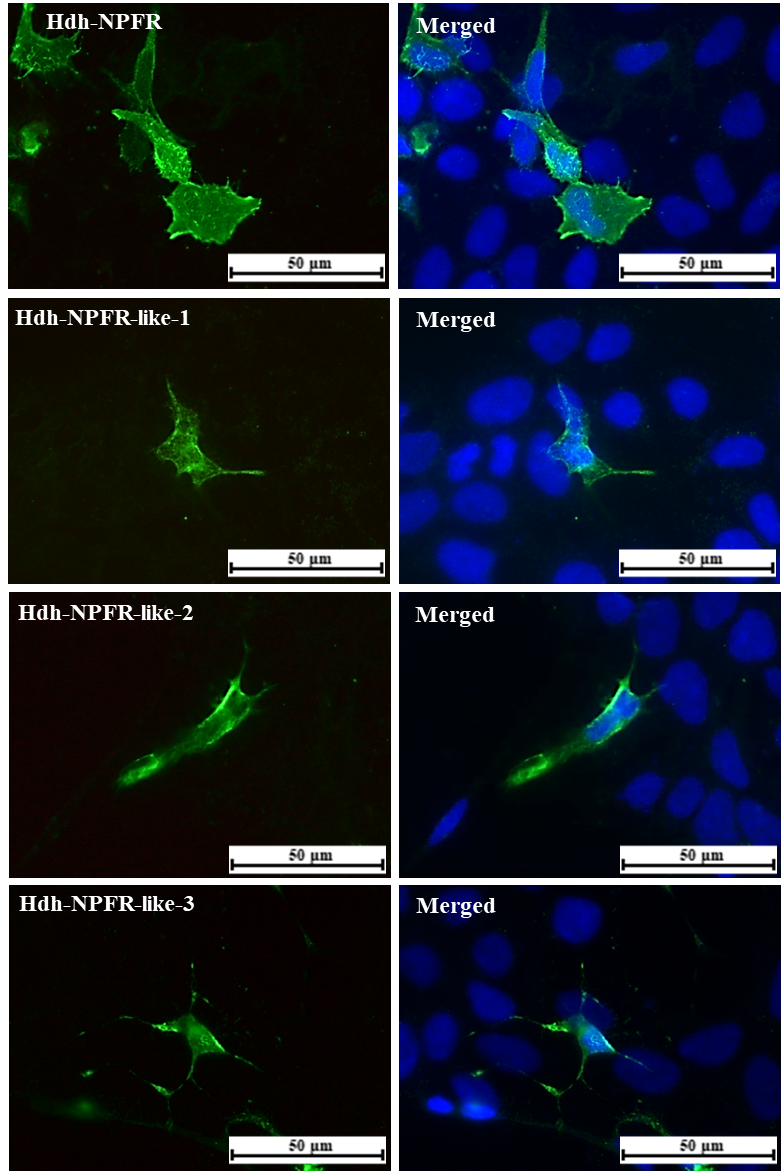
**

**Fig. S5.** Subcellular localization of Hdh-NPFR-related receptors in HEK293 cells. HA-tagged receptors were probed with a primary antibody directed against HA and labeled with an Alexa Fluor 488-conjugated secondary antibody (green); nuclei were counterstained with DAPI (blue). Scale bars = 50 μm.

**
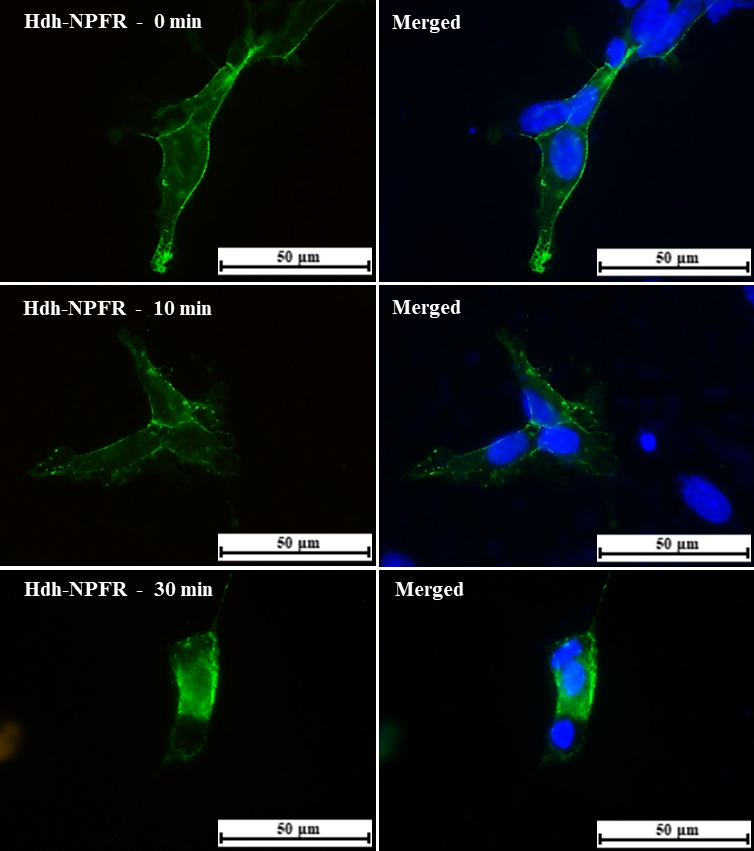
**

**Fig. S6.** Time-dependent internalization of Hdh-NPFR by Hdh-NPF treatment (10^-6^ M) in HEK293 cells. HA-tagged Hdh-NPFR was visualized by immunocytochemistry as described in the legend to **Fig. S5**. Scale bars = 50 μm.

**
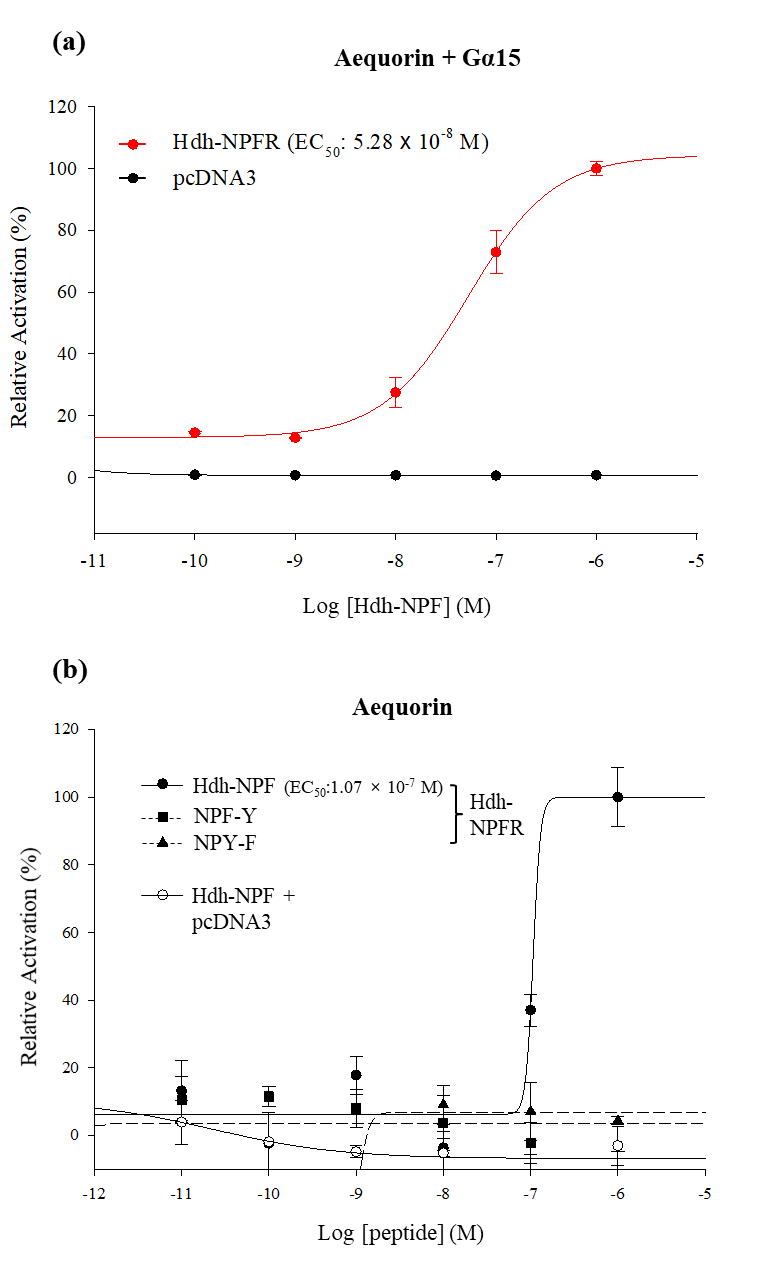
**

**Fig. S7.** Dose-response curves for intracellular Ca^2+^ in Hdh-NPFR- and aequorin-expressing CHO-K1 cells. (a) Luminescence was plotted relative to the maximal response achieved when Hdh-NPF (10^-6^ M) was applied to the Hdh-NPFR- or pcDNA3-transfected CHO-K1 cells, which were cotransfected with Gα15-expression plasmid. (b) Hdh-NPFR- or pcDNA3-transfected CHO-K1 cells were treated with various concentrations of native Hdh-NPF or mixed peptides, NPF-Y and NPY-F (see Table 1) and luminescence was plotted relative to the maximal response achieved when Hdh-NPF (10^-6^ M) was applied to the Hdh-NPFR-expressing CHO-K1 cells. Data represent mean ± SEM (n = 3 or 4).

**
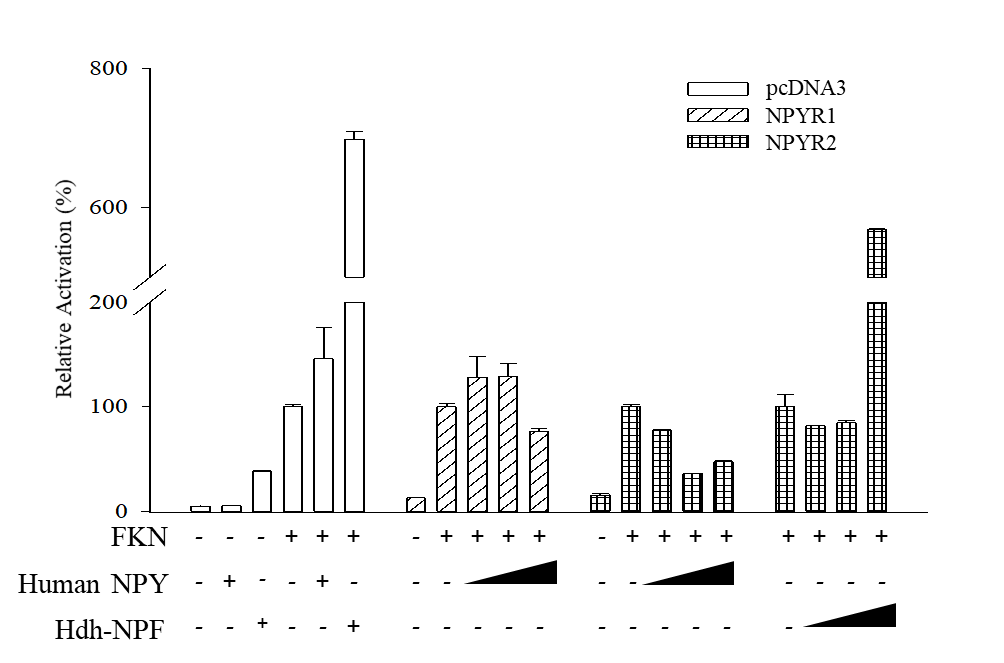
**

**Fig. S8.** Effect of human NPY and Hdh-NPF on forskolin (FKN)-stimulated CRE-Luc activities in human NPYR1- and NPYR2-expressing HEK293 cells. Intracellular cAMP accumulation was measured by CRE-Luc reporter activities in human NPYR1-, NPYR2-, or maternal plasmid pcDNA3-transfected HEK293 cells. The relative activities were determined in response to 10^-5^ M of FKN and various concentrations of human NPY or Hdh-NPF (10^-10^, 10^-8^, 10^-6^ M).

**
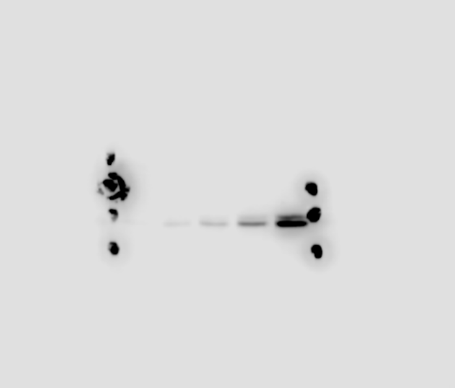
** **
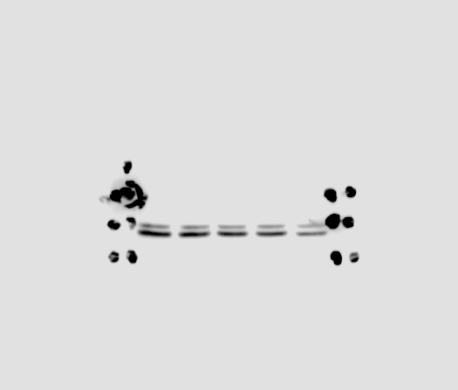
**

**Fig. 2e. upper panel with Hdh-NPFR Fig. 2e. lower panel with Hdh-NPFR**

**
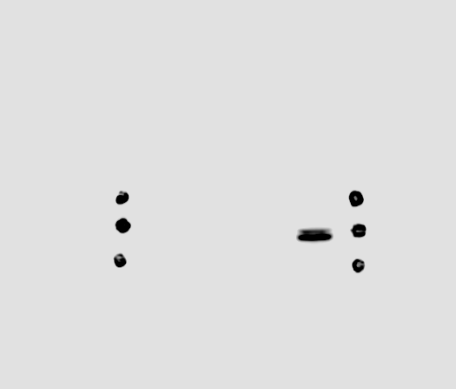

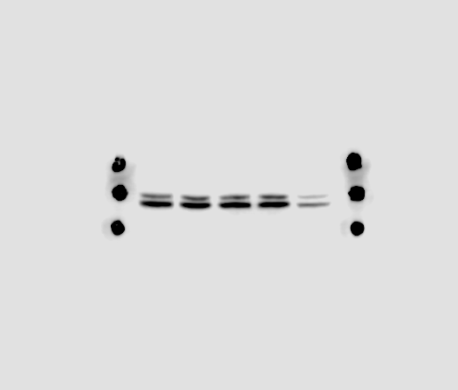
**

**Fig. 2e. upper panel with pcDNA3 Fig. 2e. lower panel with pcDNA3**

**
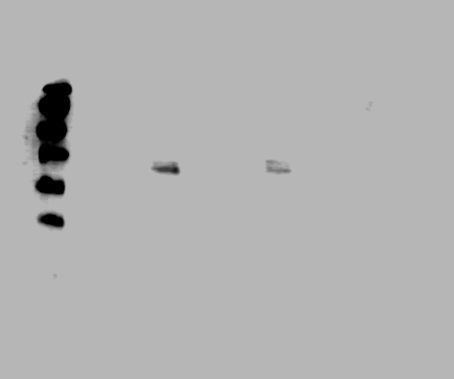
** **
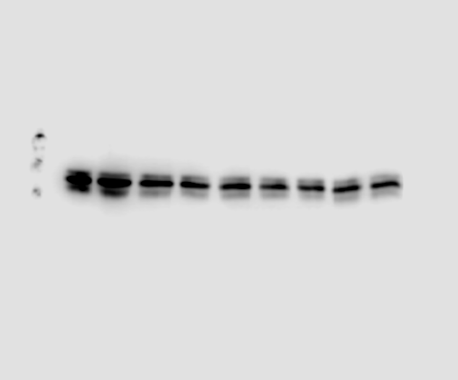
**

**Fig. 2f. upper panel with Hdh-NPFR Fig. 2f. lower panel with Hdh-NPFR**

**
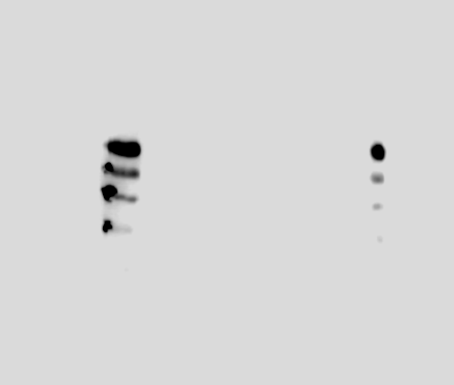
** **
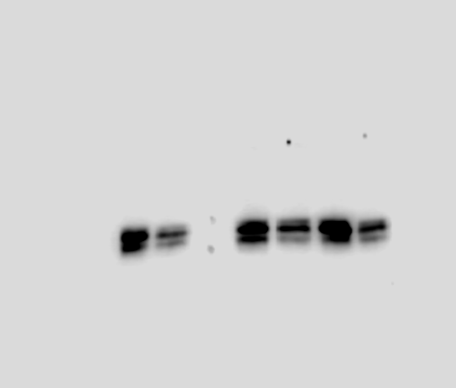
**

**Fig. 2f. upper panel with pcDNA3 Fig. 2f. lower panel with pcDNA3**

**Fig. S9.** Full-length blots for phosphorylated and total ERK1/2 in Figs. 2e and 2f. The lower panel membranes for Fig. 2e were stripped with a mild stripping condition (www.abcam.com/technical) and reprobed with a total ERK1/2 antibody. The lower panel with pcDNA3 for Fig. 2f, a molecular weight marker was loaded between second and third lanes from the left side of the image.

**
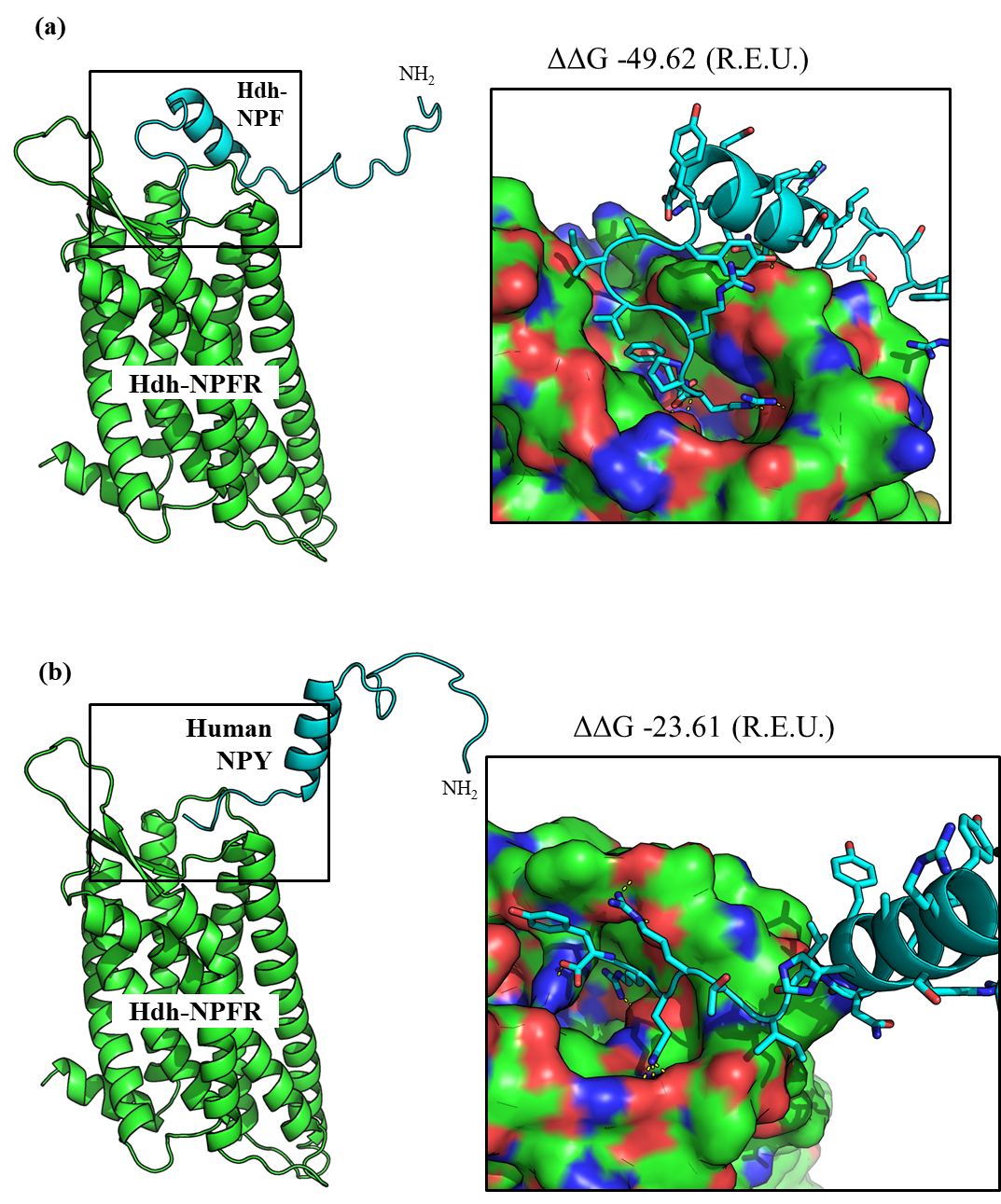
**

**Fig. S10.** *In silico* docking models of Hdh-NPF:Hdh-NPFR and human NPY:Hdh-NPFR complexes. The lowest energy docking model for (a) Hdh-NPF and (b) human NPY is shown for comparison. The binding interface is enlarged and represented as a surface view. Hdh-NPF-NPFR complex shows a stronger binding energy than human NPY for binding to Hdh-NPFR due to contact of the N-terminal NPY helix with Hdh-NPFR.

**
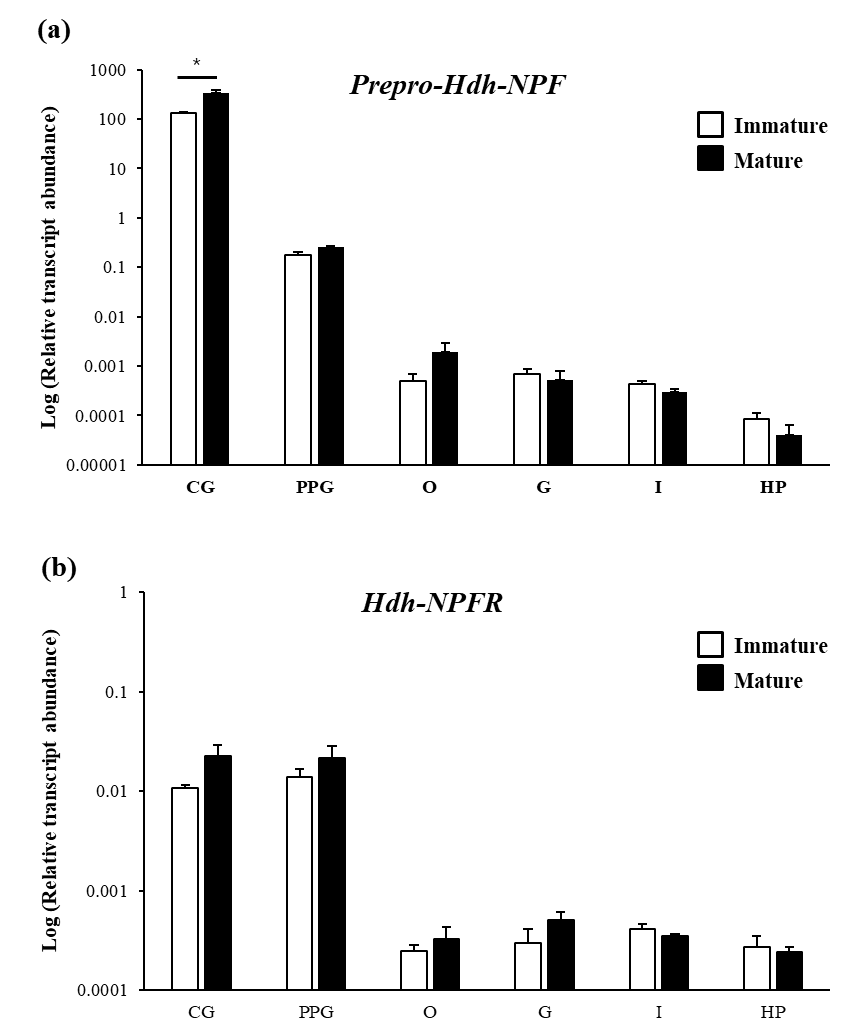
**

**Fig. S11.** Tissue distribution of *Hdh-NPF* precursor and *Hdh-NPFR* transcripts in immature and mature female abalone. (a) *Prepro-Hdh-NPF* and (b) *Hdh-NPFR* transcript levels were measured by real-time quantitative PCR. The ribosomal protein L-5 (*Hdh-RPL5*) was used as the internal control. All data represent the mean ± SEM (n = 4); *significant difference (P < 0.05) between immature and mature animals by Student’s *t*-test. Abbreviations: CG, cerebral ganglion; PPG, pleuro-pedal ganglion; O, ovary; G, gills; I, intestine; HP, hepatopancreas.


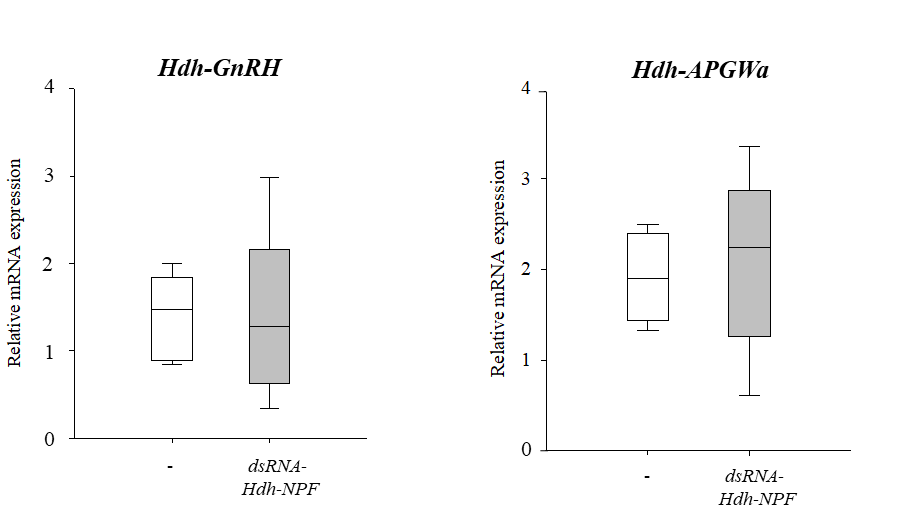


**Fig. S12.** Relative *Hdh-GnRH* and *Hdh-APGWa* transcript levels in the cerebral ganglia of abalone. The *Hdh-GnRH* and *Hdh-APGWa* transcript levels in saline- or *dsRNA-Hdh-NPF*-injected abalone were measured by RT-qPCR. The ribosomal protein L-5 (*Hdh-RPL5*) was used as the internal control. Results represent means ± SEM (n = 8). There were no statistical significances between saline- and *dsRNA-Hdh-NPF*-injected groups (P > 0.05) as observed by Mann-Whitney *U* test.

# **Supplementary Table S1.** Sources and accession numbers of the precursor sequences used for the peptide alignments and the phylogenetic analysis as shown in Fig. 1a and Supplementary Fig. S2.

| **Sequence Name** | **Species** | **Source** | **Accession number** | **Reference** |
| --- | --- | --- | --- | --- |
| *Hdh*_NPF | *Haliotis discus hannai* | NCBI | MZ027150, MZ027151 | This study |
| *L.gig*_NPF | *Lottia gigantea* | NCBI | JQ646097.1 | [77] |
| *O.bim*_NPF | *Octopus bimaculoides* | NCBI | XM_014922241.1 | - |
| *A.cal*_NPF | *Aplysia californica* | NCBI | M98854 | [78] |
| *C.gig*_NPF | *Crassostrea gigas* | NCBI | XM_011449876.2 | [79] |
| *M.yes*_NPF | *Mizuhopecten yessoensis* | NCBI | MH045240.1 | - |
| *L.sta*_NPF | *Lymnaea stagnalis* | NCBI | AJ238276 | [30] |
| *P.dum*_NPF | *Platynereis dumerilii* | NCBI | GBZT01002538.1 | - |
| *C.tel*_NPF2 | *Capitella telata* | Joint Genome Institute Genome Portal | 204022 | [42] |
| *S.med*_NPY1 | *Schmidtea mediterranea* | NCBI | GU295175 | [80] |
| *S.med*_NPY4 | *Schmidtea mediterranea* | NCBI | BK007039 |  |
| *S.med*_NPY9 | *Schmidtea mediterranea* | NCBI | BK007040 |  |
| *D.mel*_NPF | *Drosophila melanogaster* | NCBI | AF117896.1 | [81] |
| *A.aeg*_NPF | *Aedes aegypti* | NCBI | AF474405.1 | [82] |
| *B.mor*_NPF1 | *Bombyx mori* | NCBI | AB362224.1 | [83] |
| *B.mor*_NPF2 | *Bombyx mori* | NCBI | AB298926.1 |  |
| *B.flo*_NPY | *Branchiostoma floridae* | NCBI | XM_002609496.1 | [84] |
| *H.sap*_NPY | *Homo sapiens* | NCBI | NM_000905.4 | [85] |
| *D.rer*_NPY | *Danio rerio* | NCBI | NM_131074.2 | [86] |
| *G.gal*_NPY | *Gallus gallus* | NCBI | NM_205473.1 | [87] |
| *C.ele*_FLP34 | *Caenorhabditis elegans* | NCBI | NM_001383273 | [21] |
| *S.pur*_sNPF-PrRP | *Strongylocentrotus purpuratus* | NCBI | XP_001176371.1 | - |
| *B.mor*_sNPF | *Bombyx mori* | NCBI | NM_001134257.1 | [83] |
| *P.dum*_sNPF | *Platynereis dumerilii* | NCBI | JF811330.1 | [88] |
| *D.mel*_sNPF | *Drosophila melanogaster* | NCBI | NM_165316.2 | [89] |
| *A.aeg*_sNPF | *Aedes aegypti* | NCBI | XM_021844522.1 | [90] |
| *L.sta*_sNPF | *Lymnaea stagnalis* | NCBI | AY773478.1 | - |
| *A.rub_sNPF-PrRP* | *Asterias rubens* | NCBI | MK033631.1 | [22] |
| Hdh_GnRH | *Haliotis discus hannai* | NCBI | KY264019 | [73] |
| *L.sta*_GnRH | *Lymnaea stagnalis* | NCBI | QIH29241.1 | - |

**Supplementary Table S2.** NCBI accession numbers of the receptor sequences used for the phylogenetic analysis and the sequence alignment as shown in Fig. 2 and Supplementary Fig. S3.

| **Sequence Name** | **Species** | **Accession number** | **Reference** |
| --- | --- | --- | --- |
| Hdh-NPFR | *Haliotis discus hannai* | MZ014382 | This study |
| Hdh-NPFR-like-1 | *Haliotis discus hannai* | MZ014383 |  |
| Hdh-NPFR-like-2 | *Haliotis discus hannai* | MZ014384 |  |
| Hdh-NPFR-like-3 | *Haliotis discus hannai* | MZ014385 |  |
| *A.cal*_NPYR2 | *Aplysia_californica* | XP_005089627.1 | - |
| *A.cal*_NPFR-like | *Aplysia_californica* | XP_005089880.1 | - |
| *C.gig*_NPFRlike | *Crassostrea_gigas* | XP_011444490.1 | - |
| *L.gig*_NPFR | *Lottia_gigantea* | XP_009066442.1 | [91] |
| *T.cas*_NPFR | *Tribolium_castaneum* | XP_008198436.1 | - |
| *D.mel*_NPFR | *Drosophila melanogaster* | NP_001246947.1 | [51] |
| *A.aeg*_NPFR | *Aedes_aegypti* | XP_021693392.1 | [92] |
| *C.tel*_NPFR-like | *Capitella teleta* | ELT88377.1 | [91] |
| *C.ele*_NPR-12 | *Caenorhabditis elegans* | NP_001293732.1 | [93] |
| *C.ele*_NPR-11 | *Caenorhabditis elegans* | NP_508234.2 | [21] |
| *S.med*_NPYR-1 | *Schmidtea mediterranea* | ANO39130.1 | [94] |
| *S.med*_NPYR-5 | *Schmidtea mediterranea* | ANO39140.1 | [94] |
| *C.tel*_NPY/NPF-R | *Capitella teleta* | ELT98787.1 | [91] |
| *P.dum*_GPCR62 | *Platynereis_dumerili* | AKQ63068.1 | [95] |
| *G.gal*_NPYR1 | *Gallus gallus* | AAK83557.1 | [96] |
| *H.sap*_NPYR1 | *Homo sapiens* | NP_000900.1 | [14] |
| *G.gal*_NPYR4 | *Gallus gallus* | AAL84161.1 | [97] |
| *H.sap*_NPYR4 | *Homo sapiens* | NP_005963.4 | [98] |
| *D.rer*_NPYR8a | *Danio rerio* | NP_571512.1 | [99] |
| *D.rer*_NPYR8b | *Danio rerio* | NP_571511.1 | [100] |
| *D.rer*_NPYR4 | *Danio rerio* | NP_571515.1 | [99] |
| *G.gal*_NPYR5 | *Gallus gallus* | AAK83556.1 | [54] |
| *H.sap*_NPYR5 | *Homo sapiens* | NP_006165.1 | [101] |
| *D.rer*_NPYR2 | *Danio rerio* | XP_001343301.3 | [102] |
| *X.tro*_NPYR2 | *Xenopus tropicalis* | XP_004911210.1 | - |
| *G.gal*_NPYR2 | *Gallus gallus* | NP_001026299.1 | [54] |
| *H.sap*_NPYR2 | *Homo sapiens* | NP_000901.1 | [103] |
| *G.gal_*NPYR7 | *Gallus gallus* | NP_001032913.1 | [54] |
| *D.rer_*NPYR7 | *Danio rerio* | NP_001007219.1 | [104] |
| *L.sta_*GPCR105 | *Lymnaea stagnalis* | CAA57620.1 | [30] |
| *A.rub*_sNPF/PrRP-R | *Asterias rubens* | AYM55328.1 | [22] |
| *S.pur*_sNPFR/PrRP-R | *Strongylocentrotus purpuratus* | XP_003725178.2 | - |
| *C.gig*_sNPFR | *Crassostrea gigas* | XP_011451552.1 | [105] |
| *C.tel*_sNPFR | *Capitella teleta* | ELT88594.1 | [91] |
| *P.dum_*NKY-R | *Platynereis dumerilii* | AKQ63001.1 | [95] |
| *D.mel*_sNPFR-A | *Drosophila melanogaster* | NP_524176.1 | - |
| *B.mor*_sNPFR(GPR-A7) | *Bombyx_mori* | NP_001127742.1 | - |
| *B.mor*_sNPFR(GPR-A11) | *Bombyx_mori* | NP_001127708.1 | - |
| *B.mor*_sNPFR(GPR-A10) | *Bombyx_mori* | NP_001127707.1 | - |
| *A.aeg*_sNPFR | *Aedes aegypti* | AGX84998.1 | - |
| *C.ele*_sNPFR(NPR5) | *Caenorhabditis elegans* | CCD70460.1 | - |
| *C.ele*_sNPFR(NPR4) | *Caenorhabditis elegans* | NP_001300304.1 | - |
| *C.ele*_sNPFR(NPR3) | *Caenorhabditis elegans* | CAB05681.1 | - |
| *C.ele*_sNPFR(NPR1) | *Caenorhabditis elegans* | NP_501701.2 | - |
| *B.flo*_PrRP-R | *Branchiostoma floridae* | XP_002608333.1 | - |
| *H.sap*_PrRP-R | *Homo sapiens* | NP_004239.1 | - |
| *D.rer*-PrRP-R | *Danio rerio* | NP_001034615.1 | - |
| *G.gal*_PrRP-R | *Gallus gallus* | AAW30382.1 | - |
| *X.tro*_PrRP-R | *Xenopus tropicalis* | XP_002940396.1 | - |
| *S.kow*_PrRP-R1 | *Saccoglossus kowalevskii* | XP_002740053.1 | - |
| *S.kow*_PrRP-R2 | *Saccoglossus kowalevskii* | XP_006815575.1 | - |
| *S.kow*_PrRP-R3 | *Saccoglossus kowalevskii* | XP_002738225.1 | - |
| *C.ele*_Luqin-R | *Caenorhabditis elegans* | NP_001023541.1 | - |
| *A.pis*_RYamide-R1 | *Acyrthosiphon pisum* | XP_008178727.1 | - |
| *D.mel*_RYamide-R | *Drosophila melanogaster* | P25931.2 | - |
| *A.aeg*_RYamide-R | *Aedes aegypti* | AGX85003.1 | - |
| *A.cal*_Luqin-R | *Aplysia californica* | XP_012937781.1 | - |
| *L.sta*_Luqin-R | *Lymnea stagnalis* | AAB92258.1 | - |
| *O.bim*_Luqin-R | *Octopus bimaculoides* | XP_014786450.1 | - |
| *C.tel*_Luqin-R | *Capitella teleta* | ELT96089.1 | - |
| *P.dum*_Luqin-R | *Platynereis dumerilii* | KP420214.1 | - |
| *S.pur*_Luqin-R1 | *Strongylocentrotus purpuratus* | XP_783326.1,  XP_783390.1 | - |
| *A.rub*_Luqin-R1 | *Asterias rubens* | MG744509 | - |
| *S.kow*_Luqin-R | *Saccoglossus kowalevskii* | XM_002731957.1,  XM_002731958.1 | - |
| *C.int*_Tachykinin-R | *Ciona intestinalis* | XM_009863501.2 | - |
| *H.sap*_Tachykinin-R | *Homo sapiens* | AAB20303.1, NP_001049.1, NP_001050.1 | - |
| *D.mel*_Tachykinin-R | *Drosophila melanogaster* | FBtr0085507 | - |
| *O.vul*_Tachykinin-R | *Octopus vulgaris* | BAD93354.1 | - |
| *A.cal*_Tachykinin-R | *Aplysia californica* | XP_012936180.1 | - |
| *L.gig*_Tachykinin-R | *Lottia gigantea* | XP_009062052.1 | - |
| *S.pur*_Tachykinin-R | *Strongylocentrotus purpuratus* | XP_011662258.1 | - |
| *A.rub*_Tachykinin-R | *Asterias rubens* | MG744511 | - |
| *S.pur*_GPCR83 | *Strongylocentrotus purpuratus* | XP_003729750.1 | - |
| *H.sap*_GPCR83 | *Homo sapiens* | NP_057624.3 | - |
| *G.gal*_GPCR83 | *Gallus gallus* | AEO92092.1 | - |

**Supplementary references**

77. Veenstra, J. A. Neurohormones and neuropeptides encoded by the genome of *Lottia gigantea*, with reference to other mollusks and insects. *Gen. Comp. Endocrinol*. **167**, 86–103 (2010).

78. Rajpara, S. M. *et al.* Identification and molecular cloning of a neuropeptide Y homolog that produces prolonged inhibition in *Aplysia* neurons. *Neuron* **9**, 505–513 (1992).

79. Stewart, M. J. *et al.* Neuropeptides encoded by the genomes of the Akoya pearl oyster *Pinctata fucata* and Pacific oyster *Crassostrea gigas*: a bioinformatic and peptidomic survey. *BMC Genomics* **15**, 840 (2014).

80. Collins, J. J. *et al*. Genome-wide analyses reveal a role for peptide hormones in planarian germline development. *PLoS Biol*. **8**, e1000509 (2010).

81. Brown, M. R. *et al*. Identification of a *Drosophila* brain-gut peptide related to the neuropeptide Y family. *Peptides* **20**, 1035–1042 (1999).

82. Stanek D.M. *et al*. Stanek, D. M., Pohl, J., Crim, J. W. & Brown, M. R. Neuropeptide F and its expression in the yellow fever mosquito, *Aedes aegypti*. *Peptides* **23**, 1367–1378 (2002).

83. Roller, L. *et al*. The unique evolution of neuropeptide genes in the silkworm *Bombyx mori*. *Insect Biochem. Mol. Biol*. **38**, 1147–1157 (2008).

84. Putnam, N. H. *et al.* The amphioxus genome and the evolution of the chordate karyotype. *Nature* 453, 1064–1071 (2008).

85. Minth, C. D., Andrews, P. C. & Dixon, J. E. Characterization, sequence, and expression of the cloned human neuropeptide Y gene. *J. Biol. Chem*. **261**, 11974–11979 (1986).

86. Mathieu, M., Trombino, S., Argenton, F., Larhammar, D. & Vallarino, M. Developmental expression of NPY/PYY receptors zYb and zYc in zebrafish. *Ann. N. Y. Acad. Sci*. **1040**, 399–401 (2005).

87. Blomqvist, A. G., Söderberg, C., Lundell, I., Milner, R. J. & Larhammar D. Strong evolutionary conservation of neuropeptide Y: sequences of chicken, goldfish, and *Torpedo marmorata* DNA clones. *Proc. Natl. Acad. Sci. USA* **89**, 2350–2354 (1992).

88. Conzelmann, M. *et al.* Neuropeptides regulate swimming depth of *Platynereis* larvae. *Proc. Natl. Acad. Sci. USA* **108**, E1174–E1183 (2011).

89. Vanden Broeck, J. Neuropeptides and their precursors in the fruitfly, *Drosophila melanogaster*. *Peptides* **22**, 241–254 (2001).

90. Matthews, B. J., McBride, C. S., DeGennaro, M., Despo, O. & Vosshall, L. B. The neurotranscriptome of the *Aedes aegypti* mosquito. *BMC Genomics* **17**, 32 (2016).

91. Simakov, O. *et al*. Insights into bilaterian evolution from three spiralian genomes. *Nature* **493**, 526–531 (2013).

92. Liesch, J., Bellani, L. L. & Vosshall, L. B. Functional and genetic characterization of neuropeptide Y-like receptors in *Aedes aegypti*. *PLoS Negl. Trop. Dis.* **7**, e2486 (2013).

93. *C. elegans* Sequencing Consortium. Genome sequence of the nematode *C. elegans*: a platform for investigating biology. *Science* **282**, 2012–2018 (1998).

94. Saberi, A., Jamal, A., Beets, I., Schoofs, L., Newmark, P. A. GPCRs direct germline development and somatic gonad function in planarians. *PLoS Biol*. **14**, e1002457 (2016).

95. Bauknecht, P. & Jékely, G. Large-scale combinatorial deorphanization of *Platynereis* neuropeptide GPCRs. *Cell Rep*. **12**, 684–693 (2015).

96. Holmberg, S. K., Mikko, S., Boswell, T., Zoorob, R. & Larhammar, D. Pharmacological characterization of cloned chicken neuropeptide Y receptors Y1 and Y5. *J. Neurochem*. **81**, 462–471 (2002).

97. Lundell, I., Boswell, T. & Larhammar, D. Chicken neuropeptide Y-family receptor Y4: a receptor with equal affinity for pancreatic polypeptide, neuropeptide Y and peptide YY. *J. Mol. Endocrinol*. **28**, 225–235 (2002).

98. Bard, J. A., Walker, M. W., Branchek, T. A. & Weinshank, R. L. Cloning and functional expression of a human Y4 subtype receptor for pancreatic polypeptide, neuropeptide Y, and peptide YY. *J. Biol. Chem*. **270**, 26762–26765 (1995).

99. Starbäck, P. *et al*. Neuropeptide Y receptor subtype with unique properties cloned in the zebrafish: the zYa receptor. *Brain Res. Mol. Brain Res*. **70**, 242–252 (1999).

100. Lundell, I. *et al*. Cloning and characterization of a novel neuropeptide Y receptor subtype in the zebrafish. *DNA Cell Biol*. **16**, 1357–1363 (1997).

101. Hu, Y. *et al*. Identification of a novel hypothalamic neuropeptide Y receptor associated with feeding behavior. *J. Biol. Chem*. **271**, 26315–26319 (1996).

102. Fredriksson, R., Sjödin, P., Larson, E. T., Conlon, J. M. & Larhammar, D. Cloning and characterization of a zebrafish Y2 receptor. *Regul. Pept*. **133**, 32–40 (2006).

103. Herzog, H. *et al*. Cloned human neuropeptide Y receptor couples to two different second messenger systems. *Proc. Natl. Acad. Sci. USA* **89**, 5794–5798 (1992).

104. Sundström, G., Larsson, T. A., Xu, B., Heldin, J. & Larhammar, D. Interactions of zebrafish peptide YYb with the neuropeptide Y-family receptors Y4, Y7, Y8a, and Y8b. *Front. Neurosci*. **7**, 29 (2013).

105. Bigot, L. *et al*. Functional characterization of a short neuropeptide F-related receptor in a lophotrochozoan, the mollusk *Crassostrea gigas*. *J. Exp. Biol*. **217**, 2974–2982 (2014).
