# Supplementary figures and images for: NPF activates a specific NPF receptor and regulates food intake in Pacific abalone Haliotis discus hannai
Source: Sci Rep. 2021 Oct 22;11:20912. doi: 10.1038/s41598-021-00238-1 (PMC8536682; doi:10.1038/s41598-021-00238-1)

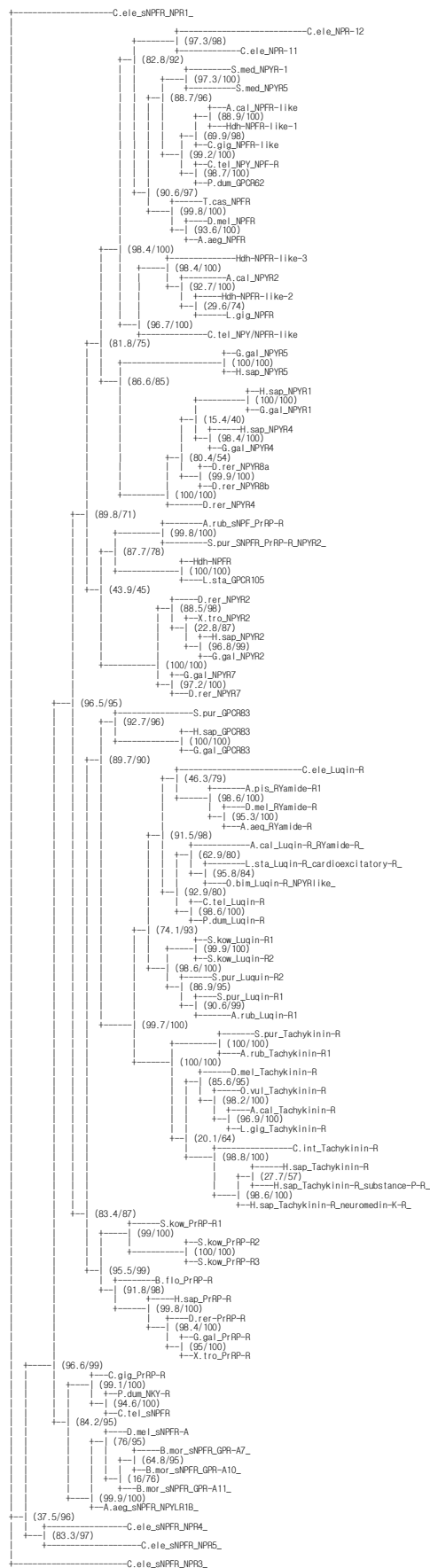

Supplement: Supplementary file 2 — Supplementary Information 2. [file 41598_2021_238_MOESM2_ESM.pdf]
